# Identification of Aortic Arch-Specific Quantitative Trait Loci for Atherosclerosis by an Intercross of DBA/2J and 129S6 Apolipoprotein E-Deficient Mice

**DOI:** 10.1371/journal.pone.0117478

**Published:** 2015-02-17

**Authors:** Yukako Kayashima, Natalia A. Makhanova, Kota Matsuki, Hirofumi Tomita, Brian J. Bennett, Nobuyo Maeda

**Affiliations:** 1 Department of Pathology and Laboratory Medicine, University of North Carolina at Chapel Hill, Chapel Hill, North Carolina, United States of America; 2 Department of Genetics, University of North Carolina at Chapel Hill, Chapel Hill, North Carolina, United States of America; University of Amsterdam Academic Medical Center, NETHERLANDS

## Abstract

The genetic background of apolipoprotein E (apoE) deficient mice influences atherosclerotic plaque development. We previously reported three quantitative trait loci (QTL), *Aath1–Aath3*, that affect aortic arch atherosclerosis independently of those in the aortic root in a cross between C57BL6 apoEKO mice (B6-apoE) and 129S6 apoEKO mice (129-apoE). To gain further insight into genetic factors that influence atherosclerosis at different vascular locations, we analyzed 335 F2 mice from an intercross between 129-apoE and apoEKO mice on a DBA/2J genetic background (DBA-apoE). The extent of atherosclerosis in the aortic arch was very similar in the two parental strains. Nevertheless, a genome-wide scan identified two significant QTL for plaque size in the aortic arch: *Aath4* on Chromosome (Chr) 2 at 137 Mb and *Aath5* on Chr 10 at 51 Mb. The DBA alleles of *Aath4* and *Aath5* respectively confer susceptibility and resistance to aortic arch atherosclerosis over 129 alleles. Both QTL are also independent of those affecting plaque size at the aortic root. Genome analysis suggests that athero-susceptibility of *Aath4* in DBA may be contributed by multiple genes, including *Mertk* and *Cd93*, that play roles in phagocytosis of apoptotic cells and modulate inflammation. A candidate gene for *Aath5* is *Stab2*, the DBA allele of which is associated with 10 times higher plasma hyaluronan than the 129 allele. Overall, our identification of two new QTL that affect atherosclerosis in an aortic arch-specific manner further supports the involvement of distinct pathological processes at different vascular locations.

## Introduction

Atherosclerosis is a systemic disease that involves multiple vascular bed, and leads to regional clinical manifestations, including stroke, myocardial infarction, abdominal aortic aneurysm, renal failure, and peripheral arterial diseases. Since multiple risk factors such as dyslipidemia and environmental determinants including diet are common to all vascular locations, it is not surprising that the severity of atherosclerosis in human patients shows a strong concordance between one location and another [1]. Nevertheless, risk factor profiles for these locations can be distinct. For example, smoking selectively increased the risk of plaques in the abdominal aorta without influencing right coronary artery lesions in 25–34 age groups [1]. Diabetes significantly increased the risk of plaques in the lower limbs, while the risk of atherosclerosis in the carotid artery was increased in hypertensive patients [2]. These heterogeneities raise the possibility that plaque development at different vascular locations involves distinct pathological processes [3].

A meta-analysis using compiled data of genome wide association studies (GWAS) from a large number of human subjects found the strongest association between coronary artery disease (CAD) and an intergenic region at chromosome 9p21, a region where only a non-coding RNA of unknown function (ANRIL) is located [4,5]. All the loci discovered so far have relatively modest effects with odds ratios <1.3, supporting the notion that CAD is generally due to “many little things” [6]. Interestingly, a majority of genes identified by a different meta-analysis of GWAS data for carotid intima/media thickness and plaque size, which are measures of subclinical atherosclerosis, were not the same as those identified by the GWAS for CAD [7]. The lack of overlapping factors between these two studies is multifactorial and could involve a combination of different clinical stages of atherosclerosis, and/or different genetic factors affecting the development of lesions at the two anatomical locations.

Established mouse models of atherosclerosis, such as apolipoprotein E knockout (*Apoe* ^*-/-*^, apoEKO) and lipoprotein receptor knockout (*Ldlr* ^*-/-*^) mice are on a C57BL6/J genetic background. In these mice, plaques develop first at the aortic root near the attachment sites of the aortic valves, a location where plaques are relatively uncommon in humans. Plaques at other locations throughout the aorta develop only as the mice age [8]. In contrast, we found that apoEKO mice on a 129S6 genetic background (129-apoE) differ from the apoEKO mice on a C57BL/6 background (B6-apoE) in atherosclerotic plaque development in a location-specific manner [9]. Plaque development at the aortic root of 129-apoE mice is slower than in B6-apoE mice. In marked contrast, however, the 129-apoE mice develop extensive plaques in the aortic arch much earlier than B6-apoE mice. Our subsequent quantitative trait loci (QTL) mapping of an F2 population of a cross between these apoEKO strains has detected two peaks (*Aath1* and *Aath2*) on Chr 1, and one peak on Chr 15 (*Aath3*), which influence the susceptibility of the aortic arch to develop lesions [10]. These three regions were distinct from QTL regions that influence aortic root plaque size, suggesting that different genetic factors are likely contributing to the strain- and location-specific differences of plaque development.

In the present study, we have carried out QTL analysis of atherosclerotic plaque size at the aortic arch in the F2 population derived from an intercross between apoEKO mice on a DBA/2J background (DBA-apoE) and those on a 129S6 background (129-apoE). We found that the loci affecting plaque size at the aortic arch between these two strains are distinct from those affecting the plaque size at their aortic root, which we reported recently [11]. With the use of genomic information on C57BL/6J, 129S5 and DBA/2J strains, and gene expression analysis of the aortic arch and peritoneal macrophage of the parental strains, we identify factors that influence early atherosclerotic plaque development in the aortic arch.

## Results

### Atherosclerotic plaque size in the aortic arch of parental, F1, and F2 apoEKO mice

**Fig 1:**
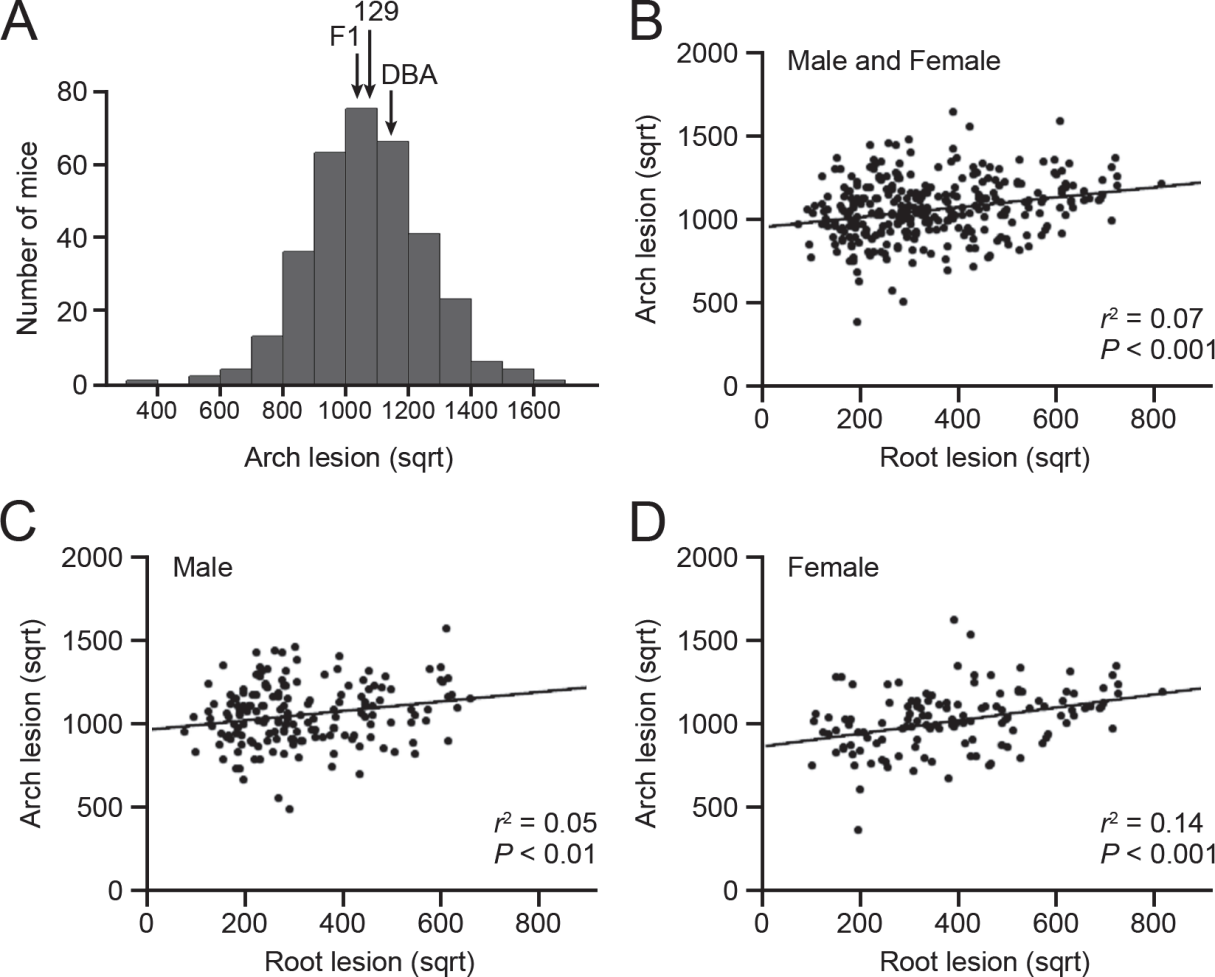
Distributions of the arch plaque size and correlations between the arch and the root plaques. (A) Histograms of square root-transformed atherosclerotic plaque size at the aortic arch in 335 F2 mice derived from 129-apoE and DBA-apoE mice. Arrows represent the positions of average plaque size for parental 129-apoE, DBA-apoE and F1 mice. (B–C) Correlations between arch and root plaque size in males and females (A), males (B), and females (C).

Atherosclerotic plaque size in the aortic arch and its branches was measured in parental 129-apoE and DBA-apoE, F1, and F2 mice as previously described (Table 1 and S1 Table) [10]. Although apoEKO mice on a DBA background develop 10 times larger plaques at their aortic root than 129-apoE mice [11], average plaque size in the aortic arch was comparable between the two strains in both males and females. In the following analysis, we used square root- transformed lesion size, which showed a normal distribution (Fig. 1A and S2 Table). A positive correlation between lesion size at the aortic arch and at the aortic root in individual F2 animals (*r* ^2^ = 0.07, *P* < 0.001) indicates that approximately 7% of the variance of plaque size at these two sites can be explained by common factors in sex-combined analysis (Fig. 1B). As shown in Fig. 1C and 1D, the correlation was weak in males (*r* ^2^ = 0.05, *P* < 0.01), but was stronger in females (*r* ^2^ = 0.14, *P* < 0.001). We observed large variations in the body weight of F2 progeny (S1A Fig.), which are weakly but positively correlated with aortic arch lesion sizes, explaining approximately 3% of the variation of arch lesion (S1B and C Fig.). No correlation was found between body weight and root lesion (S1D and E Fig.). Hypercholesterolemia is common to both strains, but the plasma lipid levels were not strong determinants of the size of aortic arch plaques (S2 Fig.).

**Table 1:** Atherosclerotic plaque size at the aortic arch area in the parental, F1, and F2 mice.

	129-apoE (n)	DBA-apoE (n)	F1 (n)	F2 (n)
Male	129.9 ± 26.3 (15)	133.0 ± 27.1 (16)	124.1 ± 33.2 (14)	121.0 ± 40.8 (191)
Female	112.7 ± 27.6 (17)^†^	129.3 ± 24.2 (16)	108.7 ± 26.3 (8)	112.5 ± 38.4 (144)

Data are shown as the mean ± SD (× 10^4^ μm^2^). ^†^ *P* < 0.05 vs. male mice within the strain.

### QTL analysis

**Fig 2:**
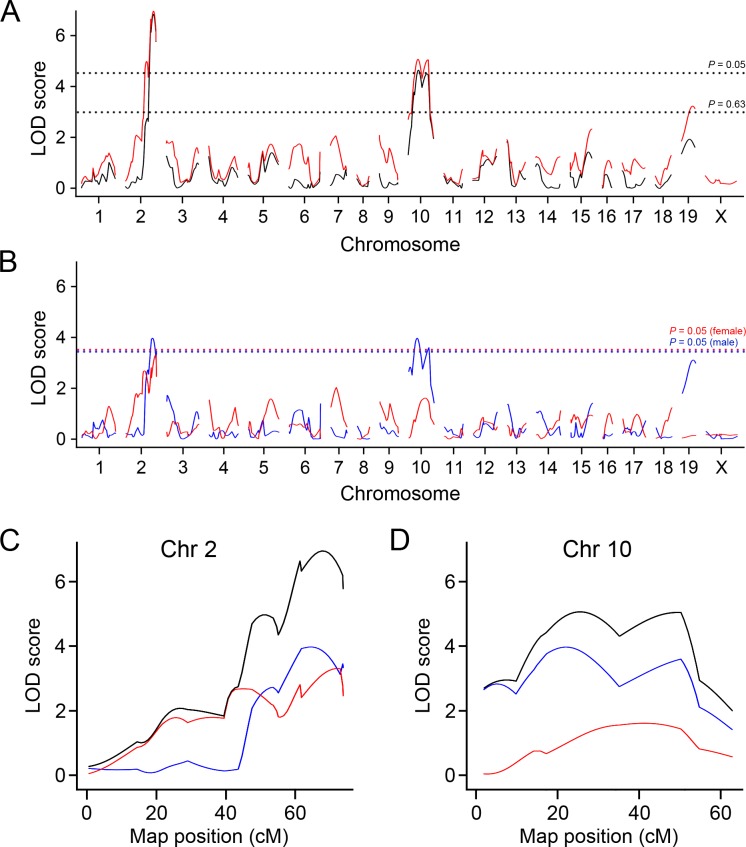
LOD curves for atherosclerotic plaque size at the arch. (A) LOD curves for the arch lesion with sex as an additive covariate (black line) and as an interactive covariate (red line). X-axis represents chromosome number and y-axis represents the LOD score. The horizontal dashed lines represent the thresholds for significant QTL (*P* = 0.05) and suggestive QTL (*P* = 0.63) in the sex-interactive model. (B) LOD curves for arch lesion in males (blue line) and females (red line). The horizontal dashed lines represent the thresholds for significant QTL (*P* = 0.05) in males (blue) and females (red). (C and D) LOD curves for the arch lesion on Chr 2 (C) and Chr 10 (D) with sex as an interactive covariate (black), in males (blue) and in females (red). X-axis represents positions on chromosome (cM) and y-axis represents the LOD score.

We performed genome-wide single scans for QTL that influence atherosclerotic plaque size (in square root) in the aortic arch and its branches using 170 SNP genotypes of 191 F2 males and 144 F2 females (Fig. 2 and S3 Fig., Table 2 and S3 Table). To account for potential gender differences and interactions, the genome scans were performed in both sexes combined using sex as an additive or interactive covariate, or separately in males and females.

**Table 2:** QTL for atherosclerosis at the aortic arch identified by genome-wide single scan.

	Chr	Peak (cM)	CI (cM)	Peak (Mb)	CI (Mb)	LOD	Significance	High Allele	Mode of Inheritance
Female and Male	2	68	61–73	137	123–148	7.0	Significant	DBA	Additive
	10	26	17–52	51	30–101	5.1	Significant	129	Additive
	19	34	17–39	39	22–46	3.2	Suggestive	129	Recessive
Male	2	65	58–74	153	132–165	4.0	Significant	DBA	Additive
	10	22	12–53	46	26–111	4.0	Significant	129	Additive
	19	33	16–39	43	25–48	3.1	Suggestive	129	Recessive
Female	2	72	36–74	132	51–134	3.3	Suggestive	DBA	Additive

Model of inheritance was determined according to allelic effect at the nearest marker of a QTL by performing linear regression using the additive and dominant/recessive models. Significant QTL are shown in bold letters. CI, 95% confidence interval.

**Fig 3:**
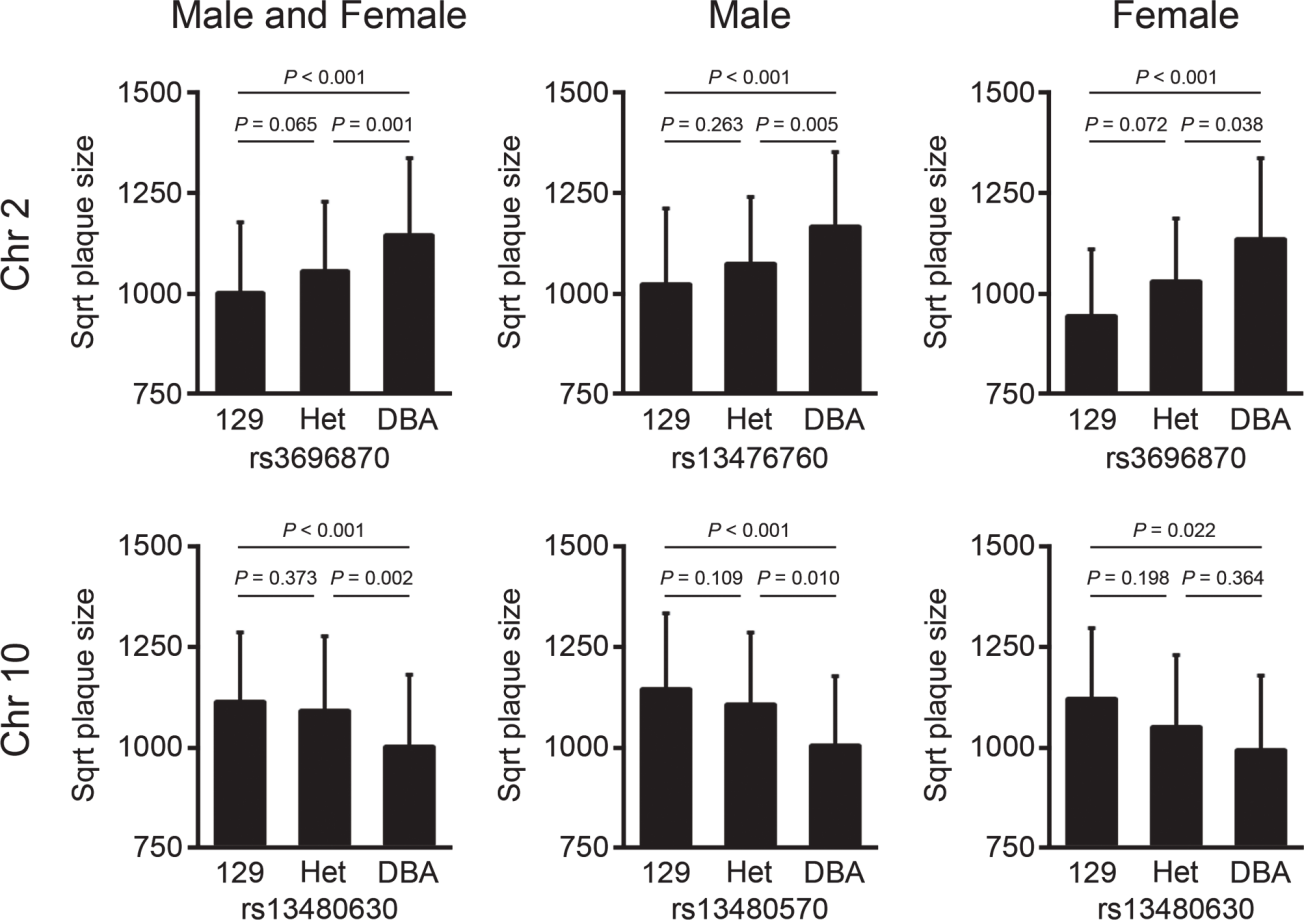
Allelic effects of QTL for atherosclerosis. Allelic distributions of the main effect QTL for arch plaques at the nearest makers to the peaks in Chr 2 and Chr 10. Atherosclerotic plaque size is indicated in square root (sqrt). Data represent the mean ± SD.

Two significant QTL for aortic arch lesion size were detected in the sex-combined analysis; one on Chr 2 (LOD = 7.0, 137 Mb, CI: 123–148 Mb) and the other on Chr 10 (LOD = 5.1, 51 Mb, CI: 30–101 Mb), which we name *Aath4* and *Aath5* respectively (Fig. 2A and Table 2). No significant differences were observed between the sex-additive and sex-interactive analyses. When two sexes were analyzed separately, the male-only analysis revealed two significant loci; one on Chr 2 (LOD = 4.0, 153 Mb) and the other on Chr 10 (LOD = 4.0, 46 Mb), and one suggestive locus on Chr 19 (LOD = 3.1, 43 Mb). In the female-only analysis, one suggestive QTL on Chr 2 (LOD = 3.3, 132 Mb) was detected (Fig. 2B and Table 2). The shape of LOD curves raised a possibility that multiple QTL might be present on Chr 2 (Fig. 2C) and Chr 10 (Fig. 2D). To search for a potential second QTL on these chromosomes, we calculated LOD score differences between the one-QTL and two-QTL models (ΔLOD) for each chromosome. The presence of an additional QTL was indicated on Chr 2 (ΔLOD = 2.3), while the evidence for a second QTL on Chr 10 was weak (ΔLOD = 1.3). To establish the relative contribution of each locus, we performed multiple regression analyses of all the detected loci together. *Aath4* and *Aath5* explained 10.7% and 5.8% of the variation in the arch lesion, respectively (S4 Table). The DBA-allele of *Aath4* on Chr 2 was associated with an increased arch plaque size over the 129-allele, while at *Aath5* on Chr 10, the 129-allele conferred increased arch plaque size relative to the DBA-allele (Fig. 3).

QTL for complex diseases such as atherosclerosis likely have epistatic interactions between loci. We therefore carried out genome-wide scans allowing for a pair-wise analysis to search for additional QTL and interactions between loci. No additional interacting loci were observed.

### Haplotype analysis and overlap with human GWAS

**Fig 4:**
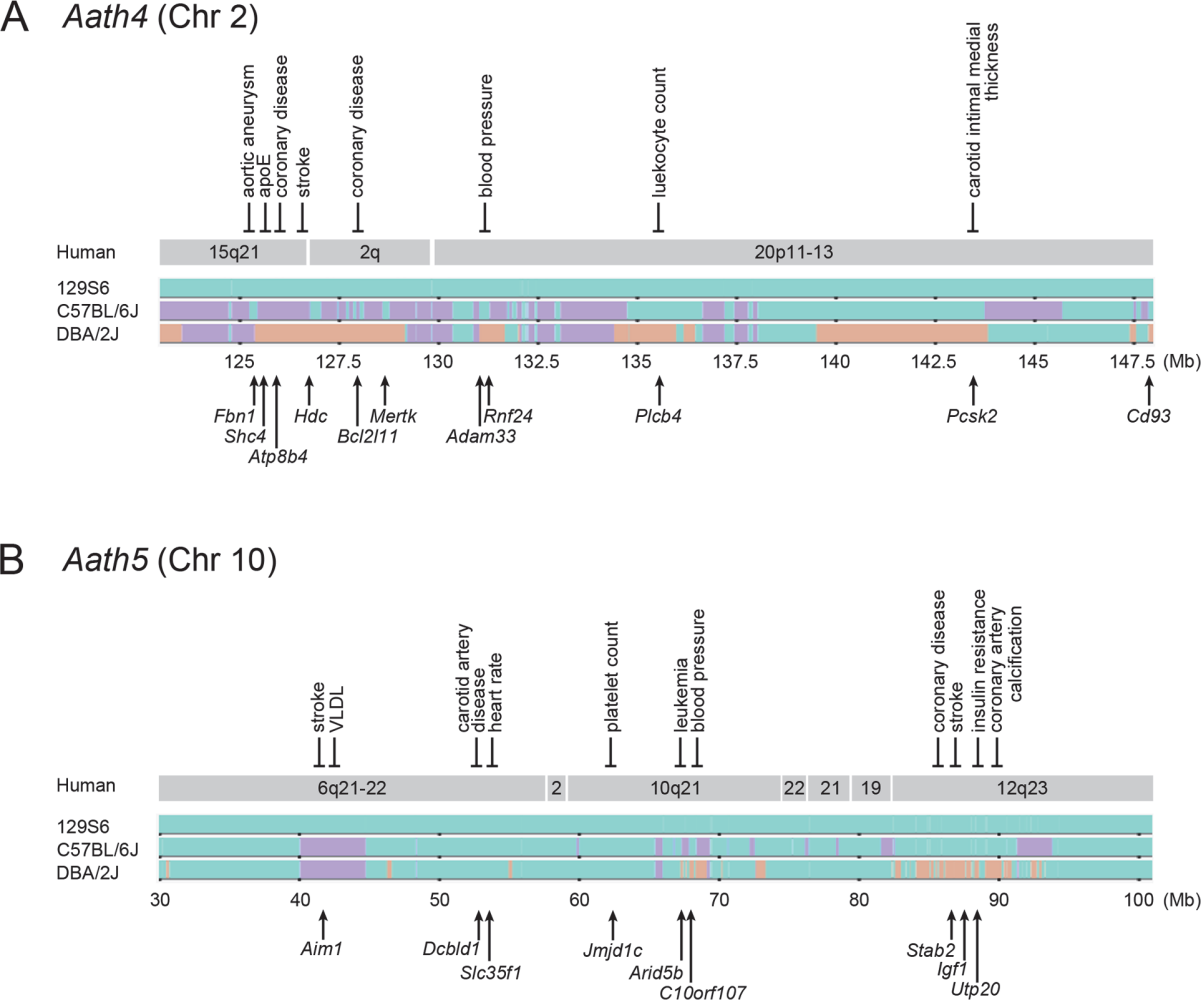
Haplotype comparison. Chromosome maps of *Aath4* on Chr 2 (A) and *Aath5* on Chr 10 (B) from UNC Mouse Phylogeny Viewer. 129 sequences and B6/DBA regions identical to 129 are shown in green; B6 sequences different from 129, and DBA sequences identical to B6 but not to 129 are shown in purple; DBA-specific sequences are shown in orange. Arrows below indicate the position of representative candidate genes. Homologous human chromosomal regions are derived from the Virtual Comparative Map (VCMap) tool. Human disease-associated regions are indicated above the human chromosomal regions.

*Aath4* for aortic arch plaques was detected at Chr 2: 137 Mb (CI: 123–148 Mb) in the current DBA-apoE × 129-apoE intercross but was not present in the previous B6-apoE ×129-apoE cross [10]. Since neither pair-wise scan nor multiple regression analysis suggested interactions of *Aath4* with any other chromosomal loci, the simplest inference is that *Aath4* lies within regions where the sequence of 129 mice differs from that of DBA mice, but is shared with that of the B6 mice. Fig. 4A illustrates the pattern of single nucleotide polymorphism (SNP) distribution in the region of Chr 2 using Mouse Phylogeny Viewer. Regions colored orange, where DBA differs from 129 and B6 and *Aath4* likely locates, are at 125–129 Mb, 131 Mb, 135 Mb, 139–143 Mb and 148–149 Mb. These regions correspond to Chr 15q21, 2q11–14 and 20p11–13 in humans, each of which contains variations associated with cardiovascular phenotypes. For example in Chr 15q21, variations in *FBN1* are associated with aortic aneurysm (*P* = 6.0 × 10^-13^), SNPs in or near *SHC4* are related to plasma apoE concentrations (*P* = 3.6 × 10^-9^), and an intronic SNPs in *ATP8B4* and *HDC* are associated with coronary disease and stroke (*P* = 5.6 × 10^-5^ and *P* = 1.3 × 10^-4^, respectively). In Chr 2q11–14, a SNP in an intron of *Bcl2l11* is related to coronary disease (*P* = 6.1 × 10^-5^). In Chr 20p11–13, a SNP in *RNF24* is associated with blood pressure (*P* = 4.0 × 10^-6^), *PLCB4* with leukocyte counts (*P* = 3.0 × 10^-10^), and *PCSK2* with carotid artery intimal medial thickness (*P* = 3.4 × 10^-6^).

*Aath5* is on Chr 10 at 51 Mb with a broad confidence interval of 30–101 Mb. Like *Aath4*, *Aath5* most likely lies within the regions where the DNA sequence of 129 mice differs from that of DBA mice, but is shared with that of the B6 mice. Haplotype comparison (Fig. 4B) shows that 129S6 and DBA/2J strains share a large portion of the *Aath5* regions on Chr 10. Regions where DBA/2J sequence differs from both 129S6 and B6 occur in four intervals: near 46 Mb, near 55 Mb, between 67.5 Mb and 69 Mb, and 84 Mb–93 Mb. These intervals correspond to human Chr 6q21–22, Chr 10q21 and Chr 12q23–24. In Chr 6q21–22, SNPs near *AIM1*–*ATG5* are associated with stroke (*P* = 9.2 × 10^-6^) and plasma VLDL concentration (*P* = 4.7× 10^-7^), those near *DCBLD1* are associated with carotid artery disease (*P* = 7.4 × 10^-8^), and a SNP in *SLC35F1* is associated with heart rate (*P* = 4.0 × 10^-10^). In Chr 10q21, variations in *JMJD1C* are related to platelet count (*P* = 2.0 × 10^-24^), those in *ARID5B* with leukemia (*P* = 7.0 × 10^-19^), and those in *C10orf107* with blood pressure (*P* = 2.0 × 10^-18^). In Chr 12q23, variations near or within *STAB2* are associated with coronary restenosis (*P* = 1.0 × 10^-7^) [12], coronary disease (*P* = 1.0 × 10^-5^) and stroke (*P* = 5.4 × 10^-4^), and those near *IGF1* are associated with insulin resistance (*P* = 2.0 × 10^-9^). Additionally, an intronic SNP in *UTP20* was associated with coronary artery calcification in the Framingham Heart Study (*P* = 7.0 × 10^-6^) [13].

These associations indicate that human chromosomal regions equivalent to both the *Aath4* and *Aath5* loci contain candidate genes with therapeutic and translational potential in human CVD and provide support for the presence of susceptible genes for atherosclerosis in the aortic arch of mice.

### Functional SNPs in the *Aath4* interval

To gain insight into candidate genes underlying each QTL, we considered single nucleotide polymorphisms (SNPs) and insertion/deletions that differ between DBA/2J and 129S5, the closest relative to 129S6 (see methods section). On the basis that SNPs are directly or by linkage responsible for the function and/or expression level of gene products, we examined potential functional consequences of these SNPs. We placed primary emphasis on SNPs that are shared by 129 and B6.

We first examined nonsynonymous SNPs with potentially damaging effects on protein structure and functions by SHIFT [14] and Polyphen-2 [15] prediction programs (S5 Table). Both programs predict a D492N substitution in fibrillin 1 (coded by *Fbn1*) to be potentially deleterious. Asp-492 is in one of the conserved EGF-like domains and may be involved in Ca^2+^ binding and/or growth factor binding to *Fbn1*, which is essential for the elastic fiber formation in aortic tissue, and mutations to which cause Marfan syndrome [16]. Similarly, a N264H substitution in one of the EGF-like domains of *Cd93* was also predicted to be deleterious. However, the functional relevance of many other substitutions including D206Y in selenocysteine binding protein 2-like (*Secisbp2l*), E33G in DTW domain containing 1(*Dtwd1*), and K177T in histidine decarboxylase (*Hdc*) is not clear, despite the fact that they involve highly conserved amino acids with charged side chains, and are predicted to be deleterious by both programs.

Some genes in the *Aath4* interval differed significantly in expression levels in aorta and/or peritoneal macrophages of parental 129S6 and DBA/2J wild type mice (S9 Table). The mRNA levels in DBA aorta of *Slc27a2*, coding for a very long chain acyl-CoA synthase was only 20% of those in 129 aorta. In contrast, expression in DBA aorta of EIA-like inhibitor of differentiation 1 (*Eid1*) and nephrocystin (*Nphp1*) were 180% and 130% of that in 129 aorta, respectively (S9 Table, *P* < 0.05).

We next searched for DBA/2J unique SNPs that are associated with variation of aortic gene expressions *in cis* or *in trans* using the available eQTL data from Hybrid Mouse Diversity Panel (HMDP) (S7 Table) [17]. In addition to *Eid1* and *Nphp1* described above, the presence of cis elements controlling expression of *Sppl2a*, *Dtwd1*, *Spef1* and *Mapre1* were suggested; although the aortic expression in parental strains did not differ significantly except for the reduced expression of the gene coding for sperm flagellar protein 1 (*Spef1*) in DBA/2J. Genes that may be controlled by the *Ath4* locus *in trans* included *Mtor* on Chr 4, *Cd24a* on Chr 10 and *Nub1* on Chr 5, but none of these genes showed differential expression in the aorta or macrophages of the parental strains.

Combining the human GWAS information, phenotypes of mouse mutants, and documented relevance to vascular health of each gene, we list 15 genes in the *Aath4* intervals in Table 3, which exhibit at least three of the six characteristics considered. Genes that exhibit at least one of the characteristics are listed in S9 Table.

**Table 3:** Haplotype analysis of *Aath4* on Chr 2.

Gene	Mb	Haplotype	Aortic expression			Macrophage expression			AA substitution	Human mutation, GWAS (-log_10_P)	KO / mutant mouse phenotype
					DBA/129	B6/129	Level	DBA/129				B6/129	Level			
Fbn1	125.1	129, B6 ≠ DBA, C3H	1.1	1.5	1684	0.9	0.2	127	A427V, **D492N**	Cholesterol (10.0), Aortic aneurysm (12.2)	Right ventricle dilation
Eid1	125.5	129, B6 ≠ DBA, C3H	1.8^a^	1.3	125	1.4	1.2	59		apoE level (8.4)	
Dtwd1	126.0	129, B6 ≠ DBA, C3H	1.1	0.9	158	1.1	0.9	170	S25P, T28A, **E33G**, D117N, E184K	Antidepressant effect (6.3)	
Hdc	126.4	129, B6 ≠ DBA, C3H	0.9	0.9	24	0.6	0.3	53	K177T	Stroke (3.9)	Abnormal mast cell morphology, Decreased plaques in *Hdc* ^*-/-*^ *Apoe* ^*-/-*^ mice
Bcl2l11	128.0	129, B6 ≠ DBA, C3H	0.7	0.8	62	0.8	2.0	262		CAD (4.2), Monocytes(6.4)	Vasculitis, Increased lymphocytes
Mertk	128.5	129, B6 ≠ DBA, C3H	1.0	1.4^a^	784	0.8	1.7	782	**W25G**, **T80E**, **S479R** and 6 others	Retinitis pigmentosa Stroke (3.3)	Abnormal retinal vascular morphology, Impaired platelet aggregation, Increased necrosis in *Mertk* ^*KD*^ *Apoe* ^*-/-*^ plaques
Adam33	130.9	129, B6, C3H ≠ DBA	0.8	1.1	176	0.7	2.7	47	K85N, T409V, R466K	BP (4.1)	Normal phenotype
Siglec1	130.9	129, B6, C3H ≠ DBA	1.5	1.4	47	0.6	2.2	787	**R162G**, and 6 others		Abnormal B cell number
Adra1d	131.4	129, B6, C3H ≠ DBA	1.3	1.1	755	1.0	1.4	16	A472V	Uric acid (7.1), Respiratory function (6.5)	Hypotension
Plcb4	135.5	129, B6, C3H ≠ DBA	1.0	0.9	1671	0.8	0.3	101		BW (5.7), Medial thickness (4.2), Leukocyte counts (9.5), HDL (4.2)	Hypoactive, Impaired coordination
Kif16b	142.4	129, B6, C3H ≠ DBA	0.8	1.0	351	1.0	1.2	436	Q759L, F825Y, **D929G**, T937N, A1005V, C1019R	Glucose(6)	Embryonic lethality, Absence of epiblast
Pcsk2	143.4	129, B6, C3H ≠ DBA	1.3	1.1	13	1.0	1.0	12		Carotid artery (4.5), Menarche (7.5)	Impaired processing of neuroendocrine hormones, Hypertension
Thbd	148.2	129, B6, C3H ≠ DBA	0.8	1.1	1110	1.0	1.0	257		Osteoporosis (12.5)	Abnormal leukocyte adhesion, Increased MI size
Cd93	148.3	129, B6, C3H ≠ DBA	1.1	1.2	489	0.9	1.3	1667	N264H		Impaired macrophage phagocytosis
Cst3	148.7	129, B6 ≠ DBA, C3H	1.2^a^	1.0	3987	1.2	1.0	3759		Kidney disease (137.7)	Thinning of tunica media and increased SM cell in atherosclerotic plaques in *Cst3* ^*-/-*^ *;Apoe* ^*-/-*^ mice

Genes with DBA-unique sequences (DBA ≠ B6, 129) within and near the interval of 123–148 Mb are shown. For each gene, expression ratios in the aorta and macrophages, expression levels in 129, amino acid (AA) differences (B6, 129-position-DBA), associations with atherosclerosis indicated by GWAS data, and phenotypes of knockout or mutant mice are shown. Ratios between two strains that show significant difference in the expression are bolded. AA substitutions that are predicted to be deleterious by SIFT program are bolded (see S5 Table).

^a^ *P* < 0.05.

### Functional SNPs in the *Aath5* interval

The same strategy was applied to identify SNPs with potential relevance to the function of the genes in the *Aath5* interval. Among nonsynonymous SNPs (S6 Table), five of 15 substitutions in Stabilin 2 (*Stab2*) of DBA were predicted to be deleterious: R151H, G864D and T1596M are located in EGF-like domains and T382S and P1086L are in fasciclin domains. Nonsynonymous SNPs in other genes were either 129 unique or predicted to be benign substitutions by both programs.

*Stab2* gene expression in the aorta was 18 fold higher in DBA than in B6 or 129 wild type mice (Table 4). Its expression in peritoneal macrophages from DBA mice was also markedly elevated. Significant but small differences in aortic expression of DBA compared to 129 mice were also observed for *Nt5dc3* (140%) and *Gnptab* (80%). In addition, eQTL data on gene expression in aortic tissues from the HMDP identified associations of SNPs that are shared between 129 and B6 but differ in DBA. These included actin related protein 6 homolog (*Actr6*), and histidine ammonia-lyase (*Hal*) on the same chromosome (S8 Table). Particularly notable is the markedly low expression of *Hal* in both aorta (20% and 30%) and macrophage (10% and 5%) of DBA and B6 mice relative to the expression in 129 tissues. None of the genes whose expression was associated *in trans* with SNPs within *Aath5* significantly differed in parental lines.

**Table 4:** Haplotype analysis of *Aath5* on Chr 10.

Gene	Mb	Haplotype	Aortic expression			Macrophage expression			AA substitution	Human mutation, GWAS (-log_10_P)	KO / mutant mouse phenotype
			DBA/129	B6/129	Level	DBA/129	B6/129	Level			
Stab2	86.3	129, B6 ≠ DBA	18.0^c^	1.4	16	11.0	1.4	18	**R151H**, K237E, V302M, T382S, I615V, I805T, **G864D**, S921N, P1086L, Q1208R, T1311S, **T1596M**, S1615L, Q1629R, V1953A	Coronary restenosis (7.0), CAD (5.0), Stroke (3.3)	Increased plasma hyaluronan
Igf1	87.3	129, B6 ≠ DBA	0.7	1.6	297	0.8	0.8	2177		Insulin resistance (8.7)	Abnormal glucose metabolism, Premature death,
Gnptab	87.8	129, B6 ≠ DBA	0.8^b^	1.0	485	0.9	1.0	1038	M206T, W785R, T817A, H994D	Mucolipidosis	Abnormal peptide metabolism, Growth retardation
Utp20	88.2	129, B6 ≠ DBA	1.0	0.8	192	1.1	0.9	325	Y1281F, N1815K	Atherosclerosis (5.2)	

Genes with DBA-unique sequences (DBA ≠ B6, 129) within and near the interval of 67.5–69 Mb and 84–93 Mb are shown. For each gene, expression ratios in the aorta and macrophages, expression levels in 129, amino acid (AA) differences (B6, 129-position-DBA), associations with atherosclerosis indicated by GWAS data, and phenotypes of knockout or mutant mice are shown. Ratios between two strains that show significant difference in the expression are bolded. AA substitutions that are predicted to be deleterious by SIFT program are bolded (see S6 Table).

^b^ *P* < 0.01

^c^ *P* < 0.001.

Nucleotide changes in non-coding exons could influence translation as well as the transcription of a gene. For example, insulin like growth factor 1 (Igf1) is produced from multiple genes, using alternative promoters and alternative splicing, which are translated and processed to produce identical Igf1 peptide of 70 amino acids [18]. There is a C to T change in the 5’UTR of *Igf1* in DBA, that results in an upstream AUG codon in some of the transcripts which could lead to a premature start of translation, resulting in an out-of-frame protein. The significance of this is not known, and plasma Igf1 levels in wild type 129 and DBA mice reported by Yuan et al. are similar (260 ± 16 ng/ml in 129S1 females versus 248 ± 10 ng/ml in DBA/2J females; 320 ± 19 ng/ml in 129S1 males versus 285 ± 10 ng/ml in DBA2/J males) [19].

Taken together, we have picked 4 potential candidate genes in the *Aath5* intervals exhibiting at least three of the six characteristics considered (Table 4). Genes that exhibit at least two of the characteristics are listed in S10 Table.

### *Stab2* genotype determines plasma hyaluronan levels

**Fig 5:**
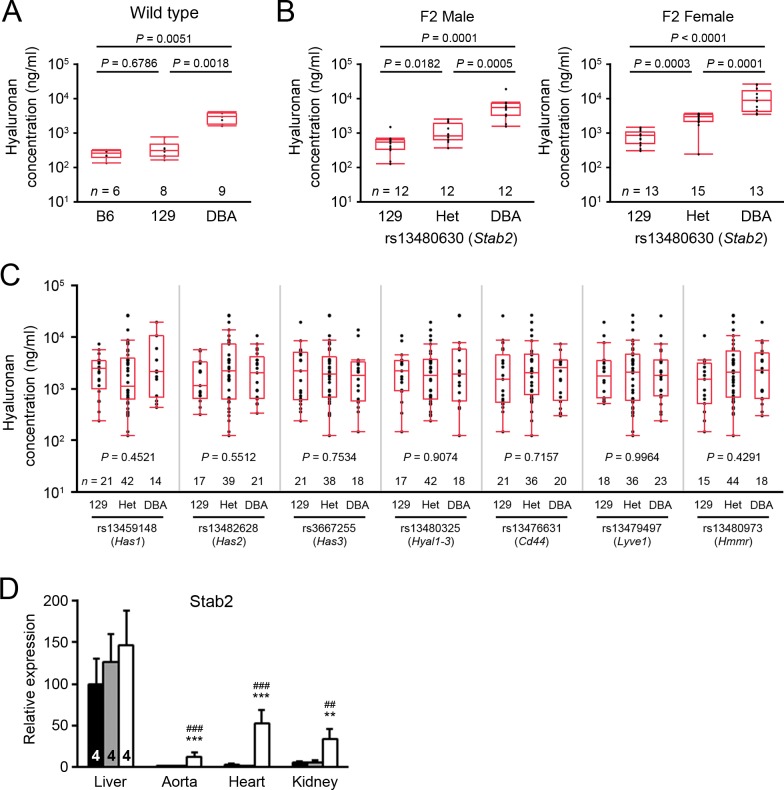
DBA-allele dependent upregulation of Stab2 and its ligand hyaluronan. (A) Plasma hyaluronan concentrations in the wild-type male C57BL/6, 129S6 and DBA2/J mice. Box-and-whisker plots: midline, median; box, 25th and 75th percentiles; whiskers, 1.5× interquartile range; dots, outliers. Numbers of mice were indicated below the plots. (B) Plasma hyaluronan concentrations in a subset of F2 males and females of 129-apoE × DBA-apoE. Mice were grouped by the genotype of rs13480630 on Chr 10, which is located near *Stab2*. Differences were compared by Kruskal–Wallis test followed by Steel-Dwass test for (A) and (B). (C) Effects of other hyaluronan metabolic loci on plasma hyaluronan concentrations. The same set of F2 population was grouped into 129-homo, Hetero and DBA-homo according to the genotype of rs13459148 on Chr 17 (near *Has1*), rs13482628 on Chr 15 (near *Has2*), rs3667255 on Chr 8 (near *Has3*), rs13480325 on Chr 9 (near *Hayl1–3*), rs13476631 on Chr 2 (near *Cd44*), rs13479497 on Chr 7 (near *Lyve1*) and rs13480973 on Chr 11 (near *Hmmr*). Kruskal–Wallis test was used for the multiple comparison. (D) Expression levels of Stab2 in various tissues from B6 (black filled bars), 129 (gray filled bars) and DBA (open bars). Data are relative to those in the B6 liver (= 100) assessed by quantitative RT-PCR. ^**^ *P* < 0.01, ^***^ *P* < 0.001 vs. B6; ^##^ *P* < 0.01, ^###^ *P* < 0.001 vs. 129 (one-way ANOVA followed by Tukey-Kramer’s HSD test). Data were shown as the mean ± SD. Sample numbers were indicated in the bars.

Since the above analysis suggested *Stabilin 2* (*Stab2*) as the most potent candidate gene for *Aath5*, we further examined whether allelic variations in the Stab2 protein of mice have effects on its function. Stab2 is a scavenger receptor that recognizes multiple ligands, including heparin, acetylated LDL, apoptotic cells and advanced glycation end products; it is also the major receptor for the clearance of hyaluronan [20–23]. We first measured plasma hyaluronan in 129S6, C57BL/6J and DBA/2J inbred mice (Fig. 5A). Strikingly, plasma hyaluronan was 10 times higher in wild type DBA mice than in B6 or 129 mice (*P* < 0.0001). Moreover, in a subset of the F2 population from the cross of DBA-apoE and 129-apoE mice, the hyaluronan levels were significantly associated with the SNP genotype on Chr 10 near *Stab2* (median values: DBA/DBA, 542.4 μg/ml; hetero, 826.9 μg/ml; 129/129, 5535.1 μg/ml in males and DBA/DBA, 482.4μg/ml; hetero, 2155.7 μg/ml; 129/129, 4171.5 μg/ml in females) (Fig. 5B). Sex had a significant effect (*P* = 0.01) and linear regression tests revealed that the DBA allele had an additive effect. Plasma hyaluronan may also be regulated by its synthesis, degradation and interaction with various receptors. However, no significant associations were observed in the same set of animals between plasma hyaluronan levels and SNP genotypes near hyaluronane synthases, *Has1*, *Has2* and *Has3*; hyaluronidases, *Hyal1–3*; or receptors, *Cd44*, *Lyve1* (lymphatic vessel endothelial hyaluronan receptor 1) and *Hmmr* (hyaluronan mediated motility receptor) (Fig. 5C).

Consistent with the previously demonstrated expression of Stab2 in liver sinusoidal endothelial cells [24], examination of the expression of *Stab2* in various tissues by real-time PCR confirmed high mRNA levels for *Stab2* in the liver, but strain difference in its liver expression was not significant (Fig. 5D). In marked contrast, while *Stab2* expression was very low in aorta, heart, and kidneys of the B6 and 129 mice, the expression was approximately 10 times higher in the respective DBA tissues (Fig. 5D). Since serum hyaluronan levels were associated with the SNP near *Stab2*, and not in other loci related to synthesis or degradation of hyaluronan, it is strongly suspected that hyaluronan clearance by Stab2 is reduced in DBA. However, elevated mRNA of *Stab2* in other tissues could compensate for local clearance of hyaluronan in DBA mice, and may also influence binding of other ligands such as heparins, modified lipoproteins and apoptotic cells. Taken together, these data support that *Stab2* is an excellent candidate gene for *Aath5*.

## Discussion

In the present study, we identified two QTL, *Aath4* and *Aath5*, which significantly affect atherosclerotic plaque size in the aortic arch area by a cross between DBA-apoE and 129-apoE.

### Overlap of *Aath4* and *Aath5* with other mouse QTL

The *Aath4* on Chr 2: 127 Mb (CI: 123–148 Mb) is close to *Ath45* on Chr 2: 162 Mb (CI: 157–165 Mb) for aortic root plaque size that we previously identified in the same cross. DBA alleles of both *Aath4* and *Ath45* associate with significantly larger plaque size than 129 alleles. However, *Ath45* was also detected in the cross between B6-apoE and 129-apoE, but QTL for aortic arch atherosclerosis was absent in this region [10]. Consequently, although closely located, the simplest inference is that the major candidate genes for *Aath4* and *Ath45* are distinct. We note, however, that these QTL regions have not been narrowed sufficiently to eliminate the possibility that multiple genes are responsible for the observed phenotypic differences with some of the genes affecting the size of plaques at both locations.

Other QTL previously mapped closely to *Aath4* include *Ath41* at 96 Mb (CI: 44–132 Mb) between apoEKO mice on BALBc background and those on B6 background [25], *Athla1* at 148 Mb (CI: 117–152 Mb) in (PERA × B6.129-*Ldlr* ^-/-^) × B6.129-*Ldlr* ^-/-^ mice [26] and *Ath28* at 179 Mb (CI: 174–179 Mb) from AKR.129-*Apoe* ^-/-^ × DBA/2.129- *Apoe* ^-/-^ mice [27]. B6 alleles of these loci confer susceptibility to atherosclerotic plaques measured in the aortic root. There is a possibility that *Aath4* is the same as the QTL at 101 Mb previously identified as a determinant of brachiocephalic artery plaque size in an F2 population between apoEKO mice on a C57BL/6J and a C3H/HeJ (C3H) background and fed a western-type high fat diet [28]. Interestingly, DBA mice share DNA variations with C3H mice within the 125–129 Mb interval, but not in the 130–143 Mb interval. Thus this haplotype block of 125–129 Mb could be the primary candidate region, although one has to be cautious since diets differ between the two studies, and *Aath4* influences plaque size not only in the brachiocephalic artery but also within the aortic arch, left carotid and left subclavian arteries (S3 Fig. and S3 Table).

While we found no significant effect of *Aath5* on the root plaque size in neither the current 129-apoE × DBA-apoE cross, nor the root or arch plaque size in the previous B6-apoE × 129-apoE cross, *Aath5* overlaps with *Ath17* [29] and *Aorls1* [30] previously reported to affect diet-induced aortic root atherosclerosis in B6 × 129 F2 females and in B6 × DBA/2 F2 females, respectively. Unlike *Aath5* in which the DBA-allele confers resistance to aortic arch atherosclerosis over the alleles in 129 and likely in B6, the DBA-allele of *Aorls1* was associated with larger plaques than the B6-allele. In addition, the B6-allele of *Ath17* confers a dominant resistance to atherosclerosis at the aortic root compared to the 129-allele. These data imply that *Aath5* is most likely a different locus from either *Ath17* or *Aorls1*, unless the same locus affects atherosclerosis development differently in a gender-, diet-, or location-dependent manner.

### Candidate genes for *Aath4* regulating inflammation

Several genes within the *Aath4* interval have been demonstrated to have potential impacts on early development of atherosclerosis. Histidine decarboxylase (Hdc) is a key enzyme for the synthesis of histamine, an important inflammatory mediator. In addition to mast cells, enterochromaffin cells and neuroendocrine cells, the expression of *Hdc* has been detected in multiple cell types including endothelial cells and macrophages. Histamine dilates micro-vessels and increases the permeability of the capillaries to white blood cells and some proteins, while it causes vasoconstriction of arteries and SMC migration [31]. Hdc is present in atherosclerotic plaques co-localizing with macrophages [32], and local production of histamine may also increase permeability to LDL and accelerate atherosclerotic plaque development. Consequently, the concept that histamine stimulates chronic inflammatory conditions in atherosclerosis has long been suspected. Supporting this view, both Hdc knockout mice and histamine receptor H1 knockout mice have been shown to develop smaller plaques in aorta when bred to apoEKO mice [33,34]. As to mouse inbred strains, Martin et al. in 1984 described that renal protein levels and enzymatic activities of Hdc in DBA/2J and C3H/HeJ mice are approximately 10 fold higher than those in other strains including C57BL6/J [35]. Martin and Bulfield further observed that the Hdc enzyme of DBA/2J binds the cofactor, pyridoxal-5’-phosphate, at a 2–3 fold higher Km than that of B6 [36]. These studies suggest that Hdc of DBA differs in both expression levels and protein structure from that of 129 and B6, possibly affecting histamine signaling in local tissues.

Interestingly, the *Aath4* interval also contains multiple genes, including *Bcl2l11* (*Bim*), *Mertk*, *Siglec1* (*Cd169*) and *Cd93*, which are involved in apoptosis and/or clearance of apoptotic cells. Efficient disposal of apoptotic cells (efferocytosis) is a crucial process in preventing cellular necrosis and release of intracellular molecules that potentially stimulate inflammation. In early atherosclerosis, efferocytosis limits plaque development by suppressing further inflammatory signaling events [37]. Mertk is a cell-surface receptor tyrosine kinase predominantly expressed in monocytic cells and functions to limit the extent of macrophage activation by inducing secretion of anti-inflammatory molecules. In a mouse model with advanced atherosclerosis, Thorp and his colleagues demonstrated that kinase defective *Mertk* promotes accumulation of apoptotic cells and expansion of necrotic cores within plaques [38]. Further investigation is required as to the functional consequence of nine amino acid differences between DBA and 129 proteins, three of which are predicted as potentially deleterious. CD93 was originally identified as a B-cell marker, but is now known to be expressed in a wide variety of cells including monocytes, activated macrophages and endothelial cells where it contributes to cell adhesion and clearance of apoptotic cells. CD93 knockout mice exhibit an impaired uptake of apoptotic cells *in vivo* [39]. The N268H substitution present in the first EGF-like domain of CD93 of DBA/2J is also present in the CD93 of NOD (non-obese diabetic) mice; in NOD mice the CD93 locus is tightly linked to diabetic susceptibility, impaired leukocyte migration and invariant natural killer cell deficiency [40]. Together, our study suggests that *Aath4* is likely to be composed of multiple genes concertedly contributing to an increased inflammatory state and accelerated atherosclerotic plaque formation in DBA-apoE mice relative to 129-apoE mice.

### *Stab2* as a candidate gene for *Aath5*

Knockout mice lacking Stab2 exhibit no specific phenotype except that they have elevated circulating hyaluronan [41,42], though the effects of the lack of Stab2 on atherosclerosis has not been evaluated. Hyaluronan is a primary constituent of the extracellular matrix and is also an abundant component of atherosclerotic plaques. Hyaluronan binds to the active form of CD44 or HMMR and mediates leukocyte adhesion and extravasation, smooth muscle cell migration and neointimal proliferation following vascular injury, suggesting that it might promote atherosclerotic plaque development [43]. Consistent with this view, Chai et al. showed that smooth muscle-specific over-expression of hyaluronan promotes the development of aortic atherosclerosis in apoEKO mice [44]. However, the contribution of hyaluronan to atherosclerosis may well be context-dependent, since hyaluronan provides viscoelastic properties to the vasculature and forms the endothelial glycocalyx, which provides protection from leukocyte adhesion. Similarly, hyaluronan can also significantly inhibit PDGF-BB-induced VSMC proliferation in culture in a dose dependent manner [45]. Nagy et al. have shown that pharmacological inhibition of hyaluronan synthesis accelerates plaque development in apoEKO mice, and that the increased atherosclerosis was accompanied by the reduced endothelial glycocalyx, endothelial dysfunction and increased prothrombotic states of the treated animals [46]. Furthermore, in cholesterol fed rabbits, Ferns et al. showed that the administration of hyaluronan significantly reduced intima-medial ratio after balloon injury; this was associated with reduced neointimal macrophage influx [47]. These observations support a possibility that the 10 times elevation of plasma hyaluronan levels is due to decreased catabolism by Stab2; this could account for the protective effects of DBA-*Aath5* on early plaque formation. However, hyaluronan is only one of many Stab2 ligands, and the effects of DBA-Stab2 on clearance of other ligands *in vivo* must be examined, since hyaluronan binding site does not appear to overlap with sites for heparin or acetylated LDL [48]. Marked increase in *Stab2* gene expression in aorta, macrophage and heart of DBA mice also indicate that local hyaluronan clearance may well be compensated for, and/or even result in an increased clearance of some other molecules thereby regulating local signaling. Clearly, further studies are necessary and we are in the process of generating congenic lines that carry the DBA allele of *Stab2* and nearby genes on the background of 129-apoE as a first step.

### Conclusions

Our QTL analysis of the cross between DBA-apoE and 129-apoE mice has identified two new loci, *Aath4* on Chr 2 and *Aath5* on Chr 10 that affect arch plaque development. Together with *Aath1* and *Aath2* on Chr 1 and *Aath3* on Chr 15, which were detected in our previous QTL analysis of a cross between B6-apoE and 129-apoE mice, there are at least five loci for arch plaque size involving 129-apoE mice. The genetic loci affecting root plaque size and arch plaque size are clearly discrete, suggesting that the pathological processes underling plaque development at the aortic arch are distinct from those at the aortic root. Our best candidate genes, *Hdc*, *Mertk* and *Cd93* for *Aath4* and *Stab2* for *Aath5*, all play pivotal roles in early processes of atherosclerosis by regulating leukocyte recruitment, macrophage activation, and inflammation. However, these processes must be common to plaque development at all vascular locations, and the major question why they appear to play roles in the development of plaques in the aortic arch but not in the aortic root remains to be answered. Differences in the local responses to these circulating factors at different vascular locations as well as the expressions of these factors within the cells in different vascular beds require further attention in future studies. Additionally, both *Aath4* and *Aath5* contain multiple potent candidates whose involvements in atherogenesis are worthy of investigation.

## Materials and Methods

### Animals

apoEKO mice on a 129S6/SvEvTac genetic background (129-apoE) were generated in our laboratory as previously described [9]. apoEKO mice on a DBA/2J background (DBA-apoE) were obtained from Jackson Laboratory (#007067, D2.129P2(B6)-Apoe^tm1Unc^/J). Male 129-apoE mice were mated to female DBA-apoE mice to generate F1 hybrids, which were intercrossed to generate F2 progeny [11]. Mice were fed regular mouse chow (Prolab IsoPro RMH 3000; Agway Inc) and handled under protocols approved by the Institutional Animal Care and Use Committees of the University of North Carolina at Chapel Hill (Protocol Number: 11–028). For euthanasia and tissue harvest, mice were fully anesthetized by inhalation of isoflurane or CO_2_ and immediately sacrificed by cervical dislocation. For atherosclerosis plaque size measurement, mice were anesthetized with an overdose of 2’2’2’-triethanol prior to perfusion of aortic trees through the heart with buffered 4% paraformaldehyde.

### Phenotyping

Atherosclerotic plaque size at the aortic arch was measured as previously described [10]. Briefly, mice at 4 months of age were perfused with 4% paraformaldehyde. Aorta with branch vessels were dissected, cleared of fat tissues, then imaged; after which atherosclerotic lesions were measured on the captured images using Image J 1.43 software (http://rsb.info.nih.gov/ij/) [10].

### DNA isolation and genotyping

Genomic DNA was isolated from livers and purified using DNeasy Tissue Kit (Qiagen, Hilden, Germany), followed by quantification using PicoGreen dsDNA Assay Kit (Molecular Probes, Eugene OR, USA). Single nucleotide polymorphism (SNP) genotyping was performed using Illumina BeadArray technology with Mouse Low Density Linkage SNP panels, which is an optimized set of 377 SNPs covering the 19 autosomes and the X chromosome and includes approximately four SNPs per each 27 Mb interval across the entire mouse genome.

### QTL analysis

QTL analyses were performed using R/qtl (v.1.24–9, http://churchill.jax.org/software/jQTLhtml) [49,50]. Square root-transformation was applied to aortic lesion size to obtain normal distribution. The main effect QTL were identified by genome-wide single-QTL scans using the ‘*scanone*’ function with standard interval mapping. Genotype probabilities between markers were computed with 1 cM intervals and with a genotyping error rate of 0.001. The significance thresholds were determined by 10,000 permutations. QTL were considered significant if LOD scores exceeded 95% (*P* < 0.05) of the permutation distribution; they were considered suggestive if the scores exceed 37% (*P* < 0.63) distribution. 95% confidence intervals (CIs) for QTL positions were calculated by Bayesian credible interval function of the software. To determine sex-specific QTL, main scans were performed with sex as an additive covariate or as an interactive covariate. We also performed QTL analysis in males and females separately. The presence of two QTL on the same chromosome was assessed by comparing two models: a model with one QTL and a model with both QTL. If the LOD score difference (ΔLOD) was greater than 2, two QTL were considered to be present on the same chromosome. For each QTL, model of inheritance was determined according to allelic effect at the nearest marker of a QTL by performing linear regression using the additive and dominant/recessive models.

Multiple-QTL models were detected by the ‘*stepwiseqtl*’ function of R/qtl, which perform a forward/backward stepwise search algorithm to select the best-fitting model. Penalties for the model comparison corresponding to α = 0.1 were calculated using the result of 1,000 permutations with the ‘*scantwo*’ function. The proportion of variance and LOD score under the model was calculated using the ‘*fitqtl*’ function. A multiple imputation was used for the analyses. All the genetic positions (cM) were converted to physical positions (Mb, NCBI Build 37) using the Mouse Map Converter (http://cgd.jax.org/mousemapconverter/).

### Microarray analysis

Aortas were dissected from 3- to 5-month-old wild type male mice. Peritoneal macrophages were isolated from 3-month-old mice of both sexes 4 days after the intraperitoneal injection of 1 ml thioglycollate. For each sample, tissues/macrophages from 4–5 mice were pooled and total RNA was extracted. For each strain, 129S6, C57BL/6J and DBA/2J, three aortic samples and one male and one female macrophage samples were subjected to Affymetrix chip analysis. Data was deposited to Gene Expression Omnibus (GEO) Database (accession number GSE53006) [11].

### Comparative genomics and haplotype analysis

Haplotype patterns of genomes in the QTL interval were analyzed using UNC Mouse Phylogeny Viewer (http://msub.csbio.unc.edu/) and Perlegen Mouse SNP Browser (http://mouse.cs.ucla.edu/perlegen/). SNPs and nucleotide sequence comparisons of mouse strains were obtained from publicly available resources (http://www.sanger.ac.uk/resources/mouse/genomes/, http://useast.ensembl.org /Mus_musculus/, and http://www.informatics.jax.org/). Sanger Genome Project includes whole genome sequence of 129S1 and 129S5 but not 129S6. We used 129S5 sequence to represent 129S6 sequence in our comparisons since they are very closely related [51]. The following characteristics were searched: (1) nonsynonymous SNPs with potentially damaging effects on protein structure and functions, (2) Genes expressed in aorta and/or peritoneal macrophages of parental C57BL6/J, DBA/2J and 129S6 wild-type mice, and differ among the strains, (3) SNPs associated with variation of gene expressions *in cis* or *in trans*. Additionally, (4) information from human GWAS, (5) phenotypes of knockout mice and (6) documented relevance to vascular health, were considered. The likelihood that an amino acid change is detrimental to a protein was examined by Sorting Intolerant From Tolerant (SIFT) program (http://sift.jcvi.org/) [14] and Polymorphism Phenotyping v2 (PolyPhen-2) program (http://genetics.bwh.harvard.edu/pph2/) [15]. Gene expression data in the aorta of mice in the Hybrid Mouse Diversity Panel (HMDP) was obtained from GEO Database (accession number GSE38120) [17]. SNPs were selected from the eQTL data of the HMDP according to the following criteria: (1) location within and near the QTL interval, (2) being associated with genes at *P* < 1.0 × 10^-6^, and (3) having nucleotide sharing pattern of DBA ≠ 129 = B6. Clusters of linked SNPs are represented by one with smallest *P* value, followed by the shortest distance to the start of the gene when it is on the same chromosome. All the genomic locations are in Mb. Information of human GWAS data was obtained from NHGRI GWAS catalog (http://www.genome.gov/gwastudies/).

### Plasma hyaluronan analysis

Plasma hyaluronan concentration was measured by Hyaluronan Quantikine ELISA Kit (R&D Systems, Minneapolis, MN) according to the manufacturer’s protocol. Plasma samples were diluted 1/40 to 1/150.

### RT-PCR

Tissues were dissected from 3-month-old male mice and stored in RNA*later* solution (Life Technologies, Carlsbad, CA). Total RNA was isolated using an ABI 6700 Automated Nucleic Acid Workstation (Applied Biosystems, Foster City, CA), and real-time quantitative RT-PCR was performed by an ABI prism 7700 sequence detection system (Applied Biosystems, Foster City, CA). The primer/probe sequences for *Stab2* were: CAGCAAGTTGATACAGGACTC (forward), TAGGCCAGAAGAGAGTGACT (reverse), and CTTGCTGAAAGTCATCACTGACCCCA (probe). *Stab2* mRNA level in each samples was normalized to that of β-actin.

### Statistical analysis

Data were analyzed using JMP software version 8.0 (SAS Institute, Cary, NC). Comparisons of multiple groups in trait values and gene expression levels were done by one-way analysis of variance (ANOVA) followed by Tukey-Kramer’s HSD test. For the analysis of SNPs and plasma hyaluronan levels, nonparametric Kruskal–Wallis (Rank-sum) test followed by Steel-Dwass test was used.

## Supporting Information

S1 Fig Correlations between root plaque size and plasma lipids. (A) Body weight in the parental 129-apoE and DBA-apoE, F1, and F2 mice. Box-and-whisker plots: midline, median; box, 25th and 75th percentiles; whiskers, 1.5× interquartile range; dots, outliers. (B–D) Correlations between body weight and arch plaque size (B and C) or root plaque size (D and E) in F2 males (B and D) and females (C and E). Plaque size was transformed to square root (sqrt).
(TIF)

S2 Fig Correlations between root plaque size and plasma lipids. Correlations of arch plaque size with total cholesterol, high-density lipoprotein (HDL) cholesterol and triglyceride in F2 males and females.
(TIF)

S3 Fig LOD curves for atherosclerotic plaque size at branches of the aortic arch. LOD curves for the lesion size at brachiocephalic artery (BCA), left common carotid artery (LCCA), left subclavian artery (LSCA) and total arch (sum of the lesions at the aortic arch and at all the branches) with sex as an interactive covariate. X-axis represents chromosome number and y-axis represents the LOD score. The horizontal dashed lines represent the thresholds for significant QTL (*P* = 0.05) and suggestive QTL (*P* = 0.63).
(TIF)

S1 Table Atherosclerotic plaque size at branches of aortic arch in the parental, F1, and F2 mice. BCA, brachiocephalic artery; LCCA, left common carotid artery; LSCA, left subclavian artery. Total arch is sum of plaques at the aortic arch and in BCA, LCCA and LSCA branches. Data are shown as the mean ± SD (× 10^4^ μm^2^). ^***^ *P* < 0.001 vs. 129-apoE mice within each sex. ^††^ *P* < 0.01 vs. male mice within the strain.
(DOC)

S2 Table Normality tests for arch plaque distribution. Distributions of arch plaque size were examined for normality by Shapiro-Wilk test before and after the square root-transformation. Null hypothesis that the data are normally distributed is not rejected when square root-transformation is applied.
(DOC)

S3 Table QTL for atherosclerosis at branches of aortic arch identified by genome-wide single scan. QTL detected by genome-wide single scan using sex as an interactive covariate are shown. % Variance shows the percentage of the total F2 phenotypic variance. Significant QTL are shown in bold letters. CI, 95% confidence interval. BCA, brachiocephalic artery; LCCA, left common carotid artery; LSCA, left subclavian artery; Total arch, sum of plaques in the Aortic arch, BCA, LCCA and LSCA.
(DOC)

S4 Table Multiple regression analyses for arch lesion in the F2 mice. df, degree of freedom; % Variance shows the percentage of the total F2 phenotypic variance.
(DOC)

S5 Table Estimated effects of amino acid substitutions in *Aath4* candidates. Effects of each amino acid substitutions in mouse proteins were predicted by SIFT (Sorting Intolerant From Tolerant) program [14]. SIFT scores show the probability that an amino acid change is damaging with a score of 0 to 1. AA substitutions with SIFT score ≤0.05 were predicted to be deleterious; substitutions with SIFT score >0.05 to be tolerated. Effects of substitutions of the residues at the equivalent position in human proteins to residues in mouse proteins were predicted by PolyPhen-2 (Polymorphism Phenotyping v2) program [15]. Where the residue in human protein differs from mouse protein, effects of substitution to both 129-type and DBA-type amino acids (shown in parentheses) were examined. PolyPhen-2 shows the probability that a mutation is damaging, ranging from 0 (benign) to 1 (damaging).----indicates no equivalent residue is present in human protein. Deleterious changes were shown in bold.
(DOC)

S6 Table Estimated effects of amino acid substitutions in *Aath5* candidates. Effects of each amino acid substitutions in mouse proteins were predicted by SIFT (Sorting Intolerant From Tolerant) program [14]. SIFT scores show the probability that an amino acid change is damaging with a score of 0 to 1. AA substitutions with SIFT score ≤0.05 were predicted to be deleterious; substitutions with SIFT score >0.05 to be tolerated. Effects of substitutions in human proteins at the equivalent residue were predicted by PolyPhen-2 (Polymorphism Phenotyping v2) program [15]. Where the residue in human protein differs from those in mice, effects of substitutions to both 129-type and DBA-type were examined. PolyPhen-2 shows the probability that a mutation is damaging, ranging from 0 (benign) to 1 (damaging). Deleterious changes were shown in bold.
(DOC)

S7 Table SNPs within the *Aath4* interval associated with gene expressions in the aorta from Hybrid Mouse Diversity Panel. Representative SNPs within and near the 123–148 Mb of Chr 2 that meet the criteria of 129 = B6 ≠ DBA and *P* < 1.0 × 10^5^ were selected from the eQTL data from the Hybrid Mouse Diversity Panel (HMDP) [17]. (-) or (+) in front of the distance indicates the position of the SNP is 5’ or 3’ respectively to the start of the gene. “trans” indicates the associated gene is on different chromosome. For each SNP, expression levels of the associated genes in the aorta (A) and macrophages (M) estimated by microarray analyses of the wild-type B6 and DBA strains relative to the expression of the 129 mice and the signal values of 129 expression are shown.
(DOC)

S8 Table SNPs within the *Aath5* interval associated with gene expressions in the aorta from Hybrid Mouse Diversity Panel. Representative SNPs within and near the 30–101 Mb intervals of Chr 10 and associated genes that meet the criteria of 129 = B6 ≠ DBA and *P* < 1.0 × 10^5^ were selected from the eQTL data from the Hybrid Mouse Diversity Panel (HMDP) [17]. For each SNP, expression levels of associated genes in the aorta (A) and macrophages (M) estimated by microarray analyses of the wild-type 129, B6 and DBA strains were indicated.
(DOC)

S9 Table Haplotype analysis of *Aath4* on Chr 2. Genes with DBA-unique sequences (DBA ≠ B6, 129) within and near the interval of 123–148 Mb are shown. For each gene, expression ratios in the aorta and macrophages, expression levels in 129, amino acid (AA) differences (B6, 129-position-DBA), associations with atherosclerosis indicated by GWAS data, and phenotypes of knockout or mutant mice are shown. Ratios between two strains that show significant difference in the expression are bolded. AA substitutions that are predicted to be deleterious by SIFT and/or Polyphen2 programs are bolded (see S5 Table). ^a^ *P* < 0.05.
(DOC)

S10 Table Haplotype analysis of *Aath5* on Chr 10. Genes with DBA-unique sequences (DBA ≠ B6, 129) within and near the interval of 30–101 Mb are shown. For each gene, expression ratios in the aorta and macrophages, expression levels in 129, amino acid (AA) differences (B6, 129-position-DBA), associations with atherosclerosis indicated by GWAS data, and phenotypes of knockout or mutant mice are shown. Ratios between two strains that show significant difference in the expression are bolded. AA substitutions that are predicted to be deleterious by SIFT and/or Polyphen2 programs are bolded (see S6 Table). ^a^ *P* < 0.05, ^b^ *P* < 0.01, ^c^ *P* < 0.001.
(DOC)
