# Supplementary figures and images for: Identification of Aortic Arch-Specific Quantitative Trait Loci for Atherosclerosis by an Intercross of DBA/2J and 129S6 Apolipoprotein E-Deficient Mice
Source: PLoS One. 2015 Feb 17;10(2):e0117478. doi: 10.1371/journal.pone.0117478 (PMC4331513; doi:10.1371/journal.pone.0117478)

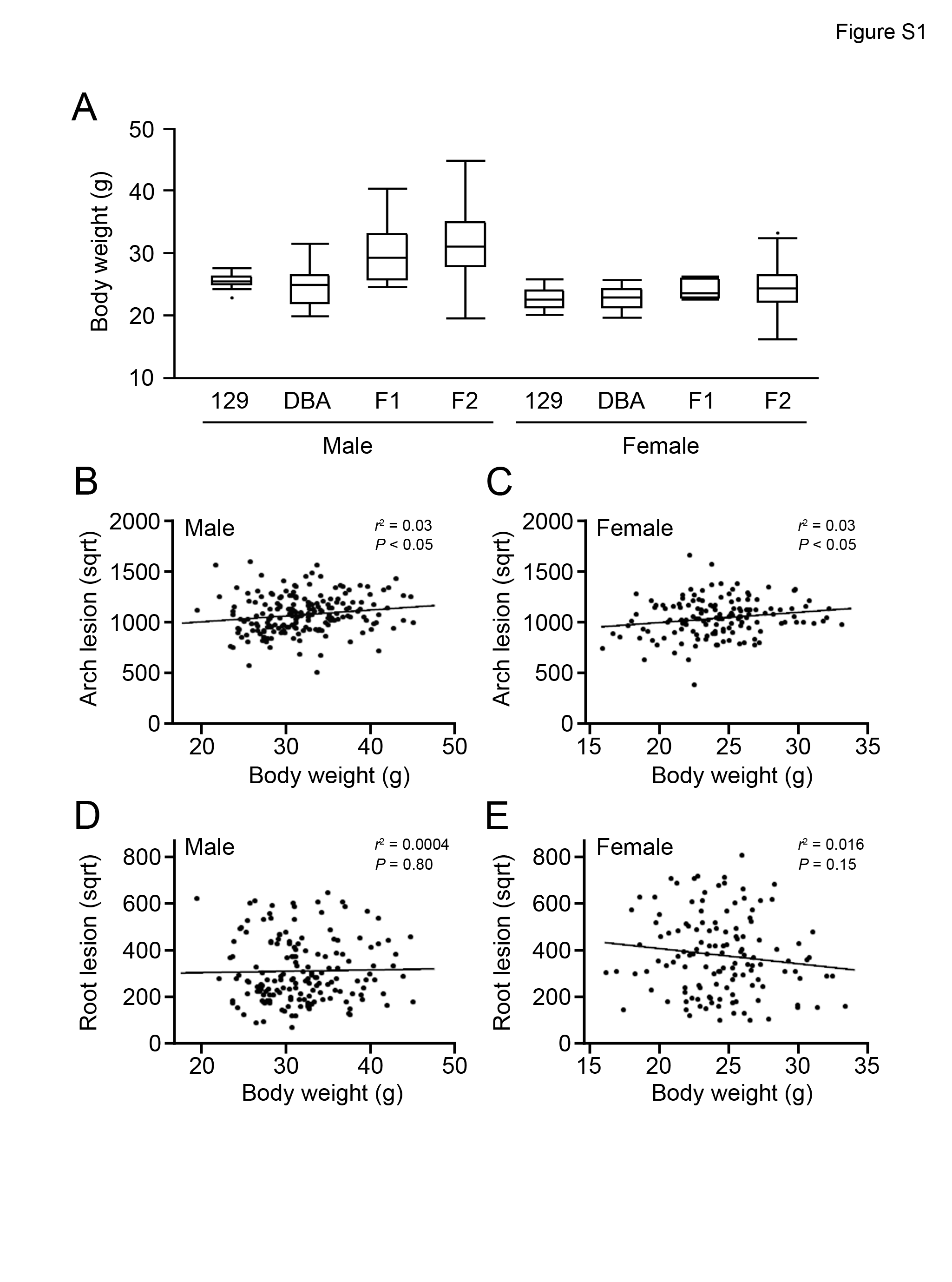

Supplement: S1 Fig — (A) Body weight in the parental 129-apoE and DBA-apoE, F1, and F2 mice. Box-and-whisker plots: midline, median; box, 25th and 75th percentiles; whiskers, 1.5× interquartile range; dots, outliers. (B–D) Correlations between body weight and arch plaque size (B and C) or root plaque size (D and E) in F2 males (B and D) and females (C and E). Plaque size was transformed to square root (sqrt). (TIF) [file pone.0117478.s001.tif]

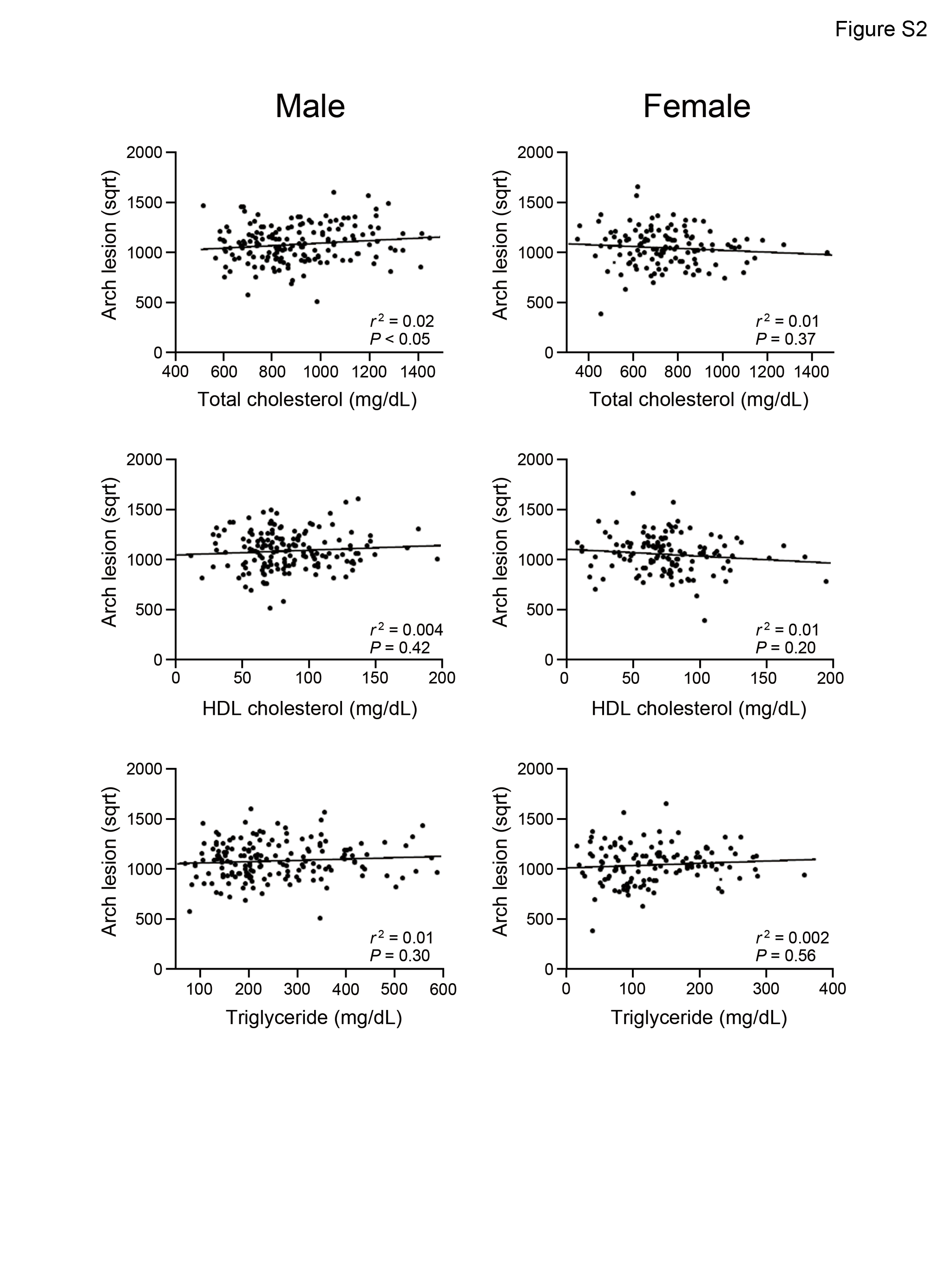

Supplement: S2 Fig — Correlations of arch plaque size with total cholesterol, high-density lipoprotein (HDL) cholesterol and triglyceride in F2 males and females. (TIF) [file pone.0117478.s002.tif]

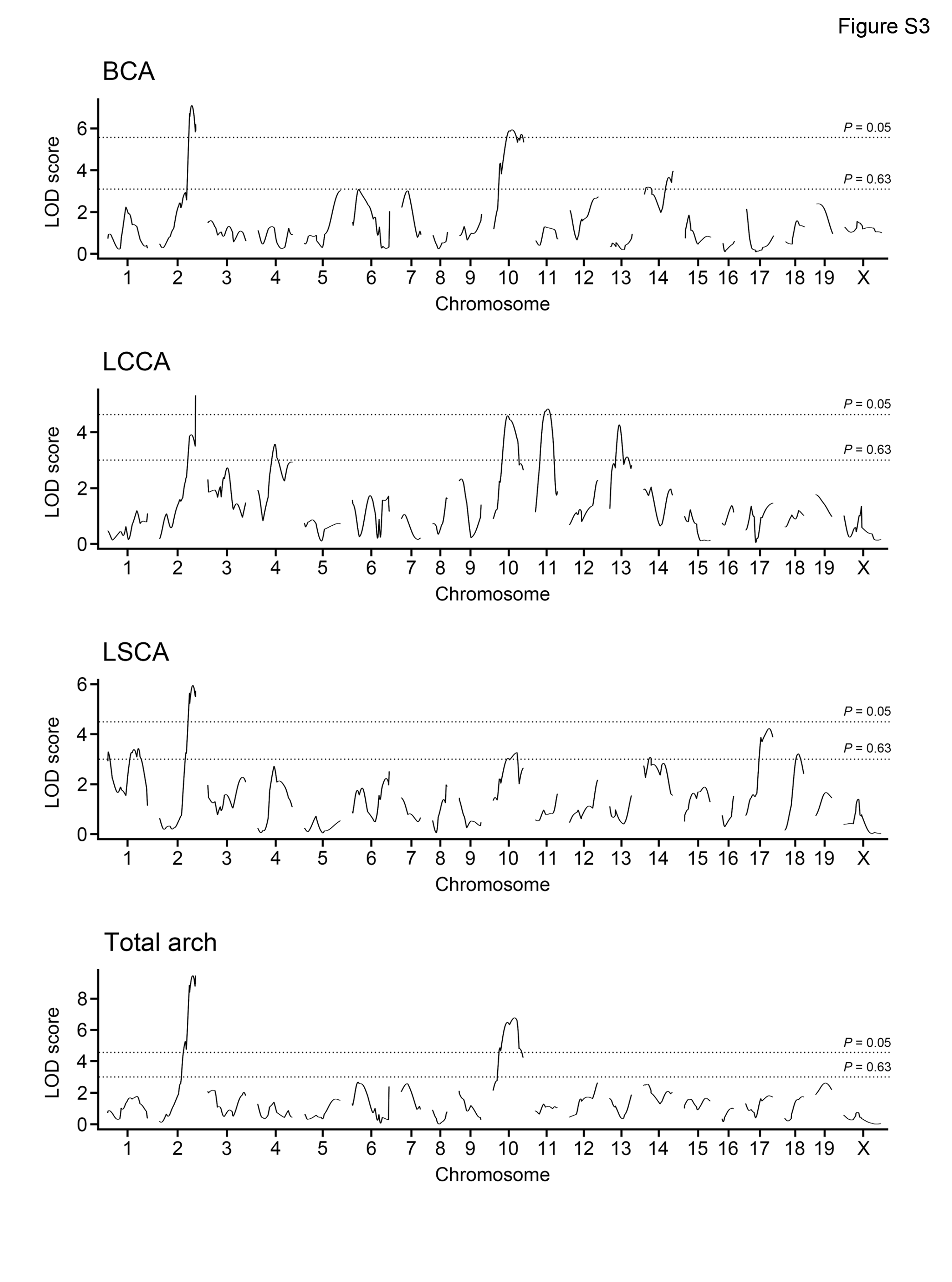

Supplement: S3 Fig — LOD curves for the lesion size at brachiocephalic artery (BCA), left common carotid artery (LCCA), left subclavian artery (LSCA) and total arch (sum of the lesions at the aortic arch and at all the branches) with sex as an interactive covariate. X-axis represents chromosome number and y-axis represents the LOD score. The horizontal dashed lines represent the thresholds for significant QTL (P = 0.05) and suggestive QTL (P = 0.63). (TIF) [file pone.0117478.s003.tif]
