# Supplementary material for: Identification of Aortic Arch-Specific Quantitative Trait Loci for Atherosclerosis by an Intercross of DBA/2J and 129S6 Apolipoprotein E-Deficient Mice
Source: PLoS One. 2015 Feb 17;10(2):e0117478. doi: 10.1371/journal.pone.0117478 (PMC4331513; doi:10.1371/journal.pone.0117478)
Supplement: S1 Table — BCA, brachiocephalic artery; LCCA, left common carotid artery; LSCA, left subclavian artery. Total arch is sum of plaques at the aortic arch and in BCA, LCCA and LSCA branches. Data are shown as the mean ± SD (× 104 μm2). *** P < 0.001 vs. 129-apoE mice within each sex. †† P < 0.01 vs. male mice within the strain. (DOC) [file pone.0117478.s004.doc]

**Table S1. Atherosclerotic plaque size at branches of aortic arch in the parental, F1, and F2 mice.**

|  |  | 129-apoE (n) | DBA-apoE (n) | F1 (n) | F2 (n) |
| --- | --- | --- | --- | --- | --- |
| BCA | Male | 48.5 ± 15.0 (15) | 44.8 ± 15.6 (16) | 52.3 ± 28.2 (14) | 42.4 ± 19.4 (186) |
|  | Female | 35.5 ± 8.4 (17)^††^ | 41.9 ± 17.0 (16) | 33.7 ± 7.7 (8) | 45.7 ± 21.2 (144) |
| LCCA | Male | 12.5 ± 5.8 (15) | 23.4 ± 8.0 (16)^***^ | 17.8 ± 12.0 (14) | 14.4 ± 9.5 (191) |
|  | Female | 11.0 ± 4.7 (17) | 30.2 ± 11.9 (16)^***^ | 12.4 ± 2.3 (8) | 13.5 ± 8.4 (144) |
| LSCA | Male | 17.0 ± 14.8 (15) | 55.9 ± 19.4 (16)^***^ | 46.5 ± 18.3 (14) | 35.0 ± 21.9 (191) |
|  | Female | 19.9 ± 14.9 (17) | 45.9 ± 21.0 (16)^***^ | 35.1 ± 14.1 (8) | 29.8 ± 18.9 (144) |
| Total arch | Male | 208 ± 26.9 (15) | 257 ± 46.7 (16)^***^ | 241 ± 73.8 (14) | 212 ± 74.6 (186) |
|  | Female | 177 ± 33.5 (17)^††^ | 247 ± 47.3 (16)^***^ | 190 ± 34.1 (8) | 202 ± 68.9 (144) |

BCA, brachiocephalic artery; LCCA, left common carotid artery; LSCA, left subclavian artery. Total arch is sum of plaques at the aortic arch and in BCA, LCCA and LSCA branches. Data are shown as the mean ± SD (× 10^4^ μm^2^). ^***^*P* < 0.001 vs. 129-apoE mice within each sex. ^††^*P* < 0.01 vs. male mice within the strain.
