# Supplementary material for: Identification of Aortic Arch-Specific Quantitative Trait Loci for Atherosclerosis by an Intercross of DBA/2J and 129S6 Apolipoprotein E-Deficient Mice
Source: PLoS One. 2015 Feb 17;10(2):e0117478. doi: 10.1371/journal.pone.0117478 (PMC4331513; doi:10.1371/journal.pone.0117478)
Supplement: S2 Table — Distributions of arch plaque size were examined for normality by Shapiro-Wilk test before and after the square root-transformation. Null hypothesis that the data are normally distributed is not rejected when square root-transformation is applied. (DOC) [file pone.0117478.s005.doc]

**Table S2.** Normality tests for arch plaque distribution.

|  | W | P |
| --- | --- | --- |
| Non transformation | 0.973 | 6.21E-06 |
| Square root-transformation | 0.995 | 0.287 |

Distributions of arch plaque size were examined for normality by Shapiro-Wilk test before and after the square root-transformation. Null hypothesis that the data are normally distributed is not rejected when square root-transformation is applied.
