# Supplementary material for: Identification of Aortic Arch-Specific Quantitative Trait Loci for Atherosclerosis by an Intercross of DBA/2J and 129S6 Apolipoprotein E-Deficient Mice
Source: PLoS One. 2015 Feb 17;10(2):e0117478. doi: 10.1371/journal.pone.0117478 (PMC4331513; doi:10.1371/journal.pone.0117478)
Supplement: S3 Table — QTL detected by genome-wide single scan using sex as an interactive covariate are shown. % Variance shows the percentage of the total F2 phenotypic variance. Significant QTL are shown in bold letters. CI, 95% confidence interval. BCA, brachiocephalic artery; LCCA, left common carotid artery; LSCA, left subclavian artery; Total arch, sum of plaques in the Aortic arch, BCA, LCCA and LSCA. (DOC) [file pone.0117478.s006.doc]

**Table S3. QTL for atherosclerosis at branches of aortic arch identified by genome-wide single scan.**

|  | Chr | Peak (cM) | CI (cM) | Peak (Mb) | CI (Mb) | LOD | Significance | High Allele | Variance (%) |
| --- | --- | --- | --- | --- | --- | --- | --- | --- | --- |
| BCA | **2** | **66** | **61-71** | **135** | **123-142** | **7.1** | **Significant** | **DBA** | **8.1** |
|  | 6 | 15 | 8-78 | 34 | 17-146 | 3.1 | Suggestive | 129 | 2.3 |
|  | **10** | **39** | **27-62** | **76** | **53-114** | **5.9** | **Significant** | **129** | **6.6** |
|  | 14 | 65 | 7-65 | 122 | 17-122 | 4.0 | Suggestive | 129 | 3.9 |
| LCCA | **2** | **74** | **61-74** | **149** | **123-149** | **5.3** | **Significant** | **DBA** | **4.7** |
|  | 4 | 36 | 25-71 | 73 | 47-139 | 3.6 | Suggestive | DBA | 2.9 |
|  | 10 | 31 | 24-51 | 60 | 45-98 | 4.6 | Suggestive | 129 | 5.3 |
|  | **11** | **40** | **29-49** | **64** | **49-82** | **4.8** | **Significant** | **DBA** | **5.6** |
|  | 13 | 21 | 10-42 | 43 | 24-81 | 4.3 | Suggestive | DBA | 3.9 |
| LSCA | 1 | 64 | 2-76 | 153 | 6-170 | 3.4 | Suggestive | DBA | 3.8 |
|  | **2** | **69** | **61-74** | **139** | **123-149** | **5.9** | **Significant** | **DBA** | **7.0** |
|  | 10 | 50 | 20-63 | 97 | 38-115 | 3.3 | Suggestive | 129 | 4.7 |
|  | 14 | 20 | 7-55 | 34 | 17-105 | 3.1 | Suggestive | 129 | 2.6 |
|  | 17 | 50 | 32-57 | 80 | 62-88 | 4.2 | Suggestive | DBA | 4.1 |
|  | 18 | 32 | 24-42 | 58 | 44-68 | 3.2 | Suggestive | DBA | 1.3 |
| Total arch | **2** | **69** | **61-74** | **139** | **125-149** | **9.5** | **Significant** | **DBA** | **12.1** |
|  | **10** | **46** | **25-52** | **91** | **50-101** | **6.8** | **Significant** | **129** | **8.9** |

QTL detected by genome-wide single scan using sex as an interactive covariate are shown. % Variance shows the percentage of the total F2 phenotypic variance. Significant QTL are shown in bold letters. CI, 95% confidence interval. BCA, brachiocephalic artery; LCCA, left common carotid artery; LSCA, left subclavian artery; Total arch, sum of plaques in the Aortic arch, BCA, LCCA and LSCA.
