# Supplementary material for: Identification of Aortic Arch-Specific Quantitative Trait Loci for Atherosclerosis by an Intercross of DBA/2J and 129S6 Apolipoprotein E-Deficient Mice
Source: PLoS One. 2015 Feb 17;10(2):e0117478. doi: 10.1371/journal.pone.0117478 (PMC4331513; doi:10.1371/journal.pone.0117478)
Supplement: S4 Table — df, degree of freedom; % Variance shows the percentage of the total F2 phenotypic variance. (DOC) [file pone.0117478.s007.doc]

**Table S4. Multiple regression analyses for arch lesion in the F2 mice.**

|  | Chromosome : cM (Mb) | df | % Variance | F value | P value |
| --- | --- | --- | --- | --- | --- |
| Male and Female | Chr 2: 31 cM (53 Mb) | 2 | 2.9 | 5.8 | 3.4 × 10^-3^ |
|  | Chr 2: 61 cM (125 Mb) | 2 | 10.7 | 21.5 | 1.7 × 10^-9^ |
|  | Chr 10: 44 cM (88 Mb) | 2 | 5.8 | 11.7 | 1.3 × 10^-5^ |
|  | Chr 19: 27 cM (32 Mb) | 2 | 1.6 | 3.2 | 4.1 × 10^-2^ |
|  | Total |  | 18.8 |  |  |
| Male | Chr 2: 35 cM (70 Mb) | 2 | 6.2 | 7.9 | 5.3 × 10^-4^ |
|  | Chr 2: 61 cM (146 Mb) | 2 | 12.9 | 16.5 | 2.6 × 10^-7^ |
|  | Chr 10: 22 cM (46 Mb) | 2 | 8.4 | 10.7 | 4.0 × 10^-5^ |
|  | Chr 19: 35 cM (45 Mb) | 2 | 4.5 | 5.7 | 3.9 × 10^-3^ |
|  | Total |  | 28.7 |  |  |
| Female | Chr 2: 25 cM (29 Mb) | 2 | 4.1 | 3.6 | 3.1 × 10^-2^ |
|  | Chr 2: 73 cM (133 Mb) | 2 | 9.4 | 8.1 | 4.6 × 10^-3^ |
|  | Chr 7: 41 cM (78 Mb) | 2 | 6.5 | 5.7 | 4.3 × 10^-3^ |
|  | Total |  | 21.1 |  |  |

df, degree of freedom; % Variance shows the percentage of the total F2 phenotypic variance.
