# Supplementary material for: Identification of Aortic Arch-Specific Quantitative Trait Loci for Atherosclerosis by an Intercross of DBA/2J and 129S6 Apolipoprotein E-Deficient Mice
Source: PLoS One. 2015 Feb 17;10(2):e0117478. doi: 10.1371/journal.pone.0117478 (PMC4331513; doi:10.1371/journal.pone.0117478)
Supplement: S5 Table — Effects of each amino acid substitutions in mouse proteins were predicted by SIFT (Sorting Intolerant From Tolerant) program [14]. SIFT scores show the probability that an amino acid change is damaging with a score of 0 to 1. AA substitutions with SIFT score ≤0.05 were predicted to be deleterious; substitutions with SIFT score >0.05 to be tolerated. Effects of substitutions of the residues at the equivalent position in human proteins to residues in mouse proteins were predicted by PolyPhen-2 (Polymorphism Phenotyping v2) program [15]. Where the residue in human protein differs from mouse protein, effects of substitution to both 129-type and DBA-type amino acids (shown in parentheses) were examined. PolyPhen-2 shows the probability that a mutation is damaging, ranging from 0 (benign) to 1 (damaging).----indicates no equivalent residue is present in human protein. Deleterious changes were shown in bold. (DOC) [file pone.0117478.s008.doc]

**Table S5. Estimated effects of amino acid substitutions in *Aath4* candidates.**

| QTL | Chr:Mb | Gene | Substitution | SIFT | Humans | PolyPhen-2 | Effect |
| --- | --- | --- | --- | --- | --- | --- | --- |
| *Aath4* | 2:125.2 | **Fbn1** | A427V | 0.53 | V-427 | 0.00 | tolerated |
|  |  |  | **D492N** | **0.01** | D-490 | **1.00** | **deleterious** |
|  | 2:125.4 | **Cep152** | M58V | 0.98 | E58 | 0.52 (M), 0.11 (V) | tolerated |
|  |  |  | S145P | 0.07 | Q157 | 0.01 (S), 0.01 (P) | tolerated |
|  |  |  | S172N | 0.22 | S183 | 0.01 | tolerated |
|  |  |  | H360Y | 1.00 | Y370 | 0.02 | tolerated |
|  |  |  | V517A | 0.30 | I527 | 0.00 (V), 0.08 (A) | tolerated |
|  |  |  | K593N | 0.77 | N605 | 0.02 | tolerated |
|  |  |  | K620Q | 1.00 | Q633 | 0.03 | tolerated |
|  |  |  | V831A | 0.89 | V798 | 0.00 | tolerated |
|  |  |  | **K979T** | 0.06 | K909 | **0.99** | **deleterious** |
|  |  |  | Y1082F | 0.77 | V1052 | 0.00 (Y). 0.00 (F) | tolerated |
|  |  |  | K1085R | 0.79 | S1055 | 0.00 (K), 0.00 (R) | tolerated |
|  |  |  | V1169 | 1.00 | ---- | ---- | tolerated |
|  |  |  | V1242S | 0.15 | V1206 | 0.02 (V), 0.73 (S) | tolerated |
|  |  |  | **R1397G** | **0.01** | K1356 | 0.00 (R), 0.00 (G) | **deleterious** |
|  |  |  | S1477F | 0.26 | S1436 | 0.00 | tolerated |
|  |  |  | P1670L | 0.81 | S1641 | 0.00 (P), 0.39 (G) | tolerated |
|  |  |  | K1680R | 0.62 | K1651 | 0.00 | tolerated |
|  |  |  | K1701R | 0.18 | K1672 | 0.00 | tolerated |
|  | 2:125.5 | Shc4 | A364T | 0.53 | H-365 | 0.00 (A), 0.00 (T) | tolerated |
|  | 2:125.7 | **Secisbp2l** | **D206Y** | **0.03** | D-206 | **1.00** | **deleterious** |
|  |  |  | A601T | 0.67 | T-623 | 0.00 | tolerated |
|  | 2:125.7 | **Galk2** | P117R | 0.53 | T-128 | 0.00 (P), 0.01 (R) | tolerated |
|  |  |  | M219V | 1.00 | V-230 | 0.12 | tolerated |
|  |  |  | **R299K** | **0.02** | E-310 | 0.00 (R), 0.00 (K) | **deleterious** |
|  | 2:126.0 | **Dtwd1** | S25P | 0.23 | S-25 | 0.00 | tolerated |
|  |  |  | T28A | 0.82 | T-28 | 0.00 | tolerated |
|  |  |  | **E33G** | **0.03** | E-33 | **0.80** | **deleterious** |
|  |  |  | D117N | 0.26 | E-117 | 0.00 (D), 0.01 (N) | tolerated |
|  |  |  | E184K | 0.35 | Q-184 | 0.00 (E), 0.01 (K) | tolerated |
|  | 2:125.2 | Atp8b4 | I424S | 0.55 | D-422 | 0.00 (I), 0.00 (S) | tolerated |
|  |  |  | N540S | 0.12 | N538 | 0.01 | tolerated |
|  |  |  | S726N | 0.34 | N724 | 0.00 | tolerated |
|  |  |  | D737E | 1.00 | E735 | 0.00 | tolerated |
|  |  |  | A746S | 0.93 | S744 | 0.00 | tolerated |
|  | 2:126.4 | Slc27a2 | L529I | 1.00 | I529 | 0.00 | tolerated |
|  |  |  | A547S | 0.46 | A540 | 0.00 | tolerated |
|  | 2:126.4 | **Hdc** | **K177T** | **0.00** | K170 | **0.75** | **deleterious** |
|  | 2:126.5 | Usp8 | K162R | 0.13 | K151 | 0.00 | tolerated |
|  |  |  | A276T | 0.33 | A265 | 0.00 | tolerated |
|  |  |  | T360A | 1.00 | A349 | 0.00 | tolerated |
|  |  |  | E381G | 0.34 | E370 | 0.00 | tolerated |
|  |  |  | P587L | 0.27 | S584 | 0.00 | tolerated |
|  | 2:126.6 | Usp50 | T128A | 0.11 | M127 | 0.00 (T), 0.00 (A) | tolerated |
|  | 2:126.8 | **Ap4e1** | **Q448H** | **0.01** | Q448 | **0.85** | **deleterious** |
|  |  |  | S809G | 0.23 | S809 | 0.04 | tolerated |
|  |  |  | F847L | 0.77 | F962 | 0.01 | tolerated |
|  |  |  | T1051A | 0.97 | K1070 | 0.00 (T), 0.02 (T) | tolerated |
|  | 2:126.9 | Blvra | S2N | 0.92 | N2 | 0.22 | tolerated |
|  | 2:126.9 | Ncaph | A568T | 0.55 | L575 | 0.00 (A), 0.00 (T) | tolerated |
|  |  |  | S114T | 1.00 | T119 | 0.13 | tolerated |
|  | 2:127.2 | **Dusp2** | **A103T** | 0.47 | A99 | **0.95** | **deleterious** |
|  | 2:127.2 | Astl | R97Q | 1.00 | Q118 | 0.00 | tolerated |
|  |  |  | Q364K | 0.74 | Q385 | 0.00 | tolerated |
|  |  |  | P397L | 0.09 | A414 | **0.99 (P), 0.83 (L)** | **deleterious** |
|  | 2:127.3 | **Gpat2** | **A238V** | 0.14 | A241 | **1.00** | **deleterious** |
|  | 2:127.3 | Fahd2a | V16F | 0.23 | A16 | 0.00 (V), 0.15 (F) | tolerated |
|  | 2:127.4 | **Prom2** | I156T | 0.39 | V156 | 0.00 (I), 0.00 (T) | tolerated |
|  |  |  | G316S | 0.48 | R315 | 0.00 (G), 0000 (S) | tolerated |
|  |  |  | **R411C** | **0.01** | S410 | 0.00 (R), **0.99 (C)** | **deleterious** |
|  |  |  | **V495A** | 1.00 | A498 | **0.99** | **deleterious** |
|  |  |  | S588N | 1.00 | S587 | 0.00 | tolerated |
|  |  |  | R753H | 0.28 | R752 | 0.01 | tolerated |
|  | 2:127.4 | **Zfp661** | **A2S** | 0.06 | A2 | **1.00** | **deleterious** |
|  |  |  | I74M | 0.31 | M74 | 0.00 | tolerated |
|  |  |  | G368S | 0.88 | S372 | 0.00 | tolerated |
|  | 2:127.4 | Mrps5 | V68A | 1.00 | A66 | 0.00 | tolerated |
|  |  |  | V75I | 0.70 | I73 | 0.00 | tolerated |
|  | 2:127.5 | Mal | V32I | 0.74 | I32 | 0.00 | tolerated |
|  | 2:127.6 | **Nphp1** | D217E | 1.00 | E215 | 0.00 | tolerated |
|  |  |  | **L540M** | **0.01** | L582 | **1.00** | **deleterious** |
|  |  |  | S590A | 1.00 | A632 | 0.20 | tolerated |
|  |  |  | A662T | 0.54 | A704 | 0.716 | tolerated |
|  | 2:127.6 | Bub1 | P153S | 0.80 | P153 | 0.32 | tolerated |
|  |  |  | T683I | 0.48 | I707 | 0.00 | tolerated |
|  |  |  | V712I | 0.34 | V738 | 0.01 | tolerated |
|  | 2:127.7 | Acoxl | R55K | 0.77 | T29 | 0.00 (R), 0.00 (K) | tolerated |
|  |  |  | M281T | 1.00 | T255 | 0.67 | tolerated |
|  |  |  | V323L | 1.00 | L297 | 0.05 | tolerated |
|  |  |  | S349L | 0.05 | S323 | 0.00 | tolerated |
|  |  |  | D389G | 0.71 | ---- | ---- | tolerated |
|  | 2:128.5 | **Mertk** | **W25G** | **0.05** | R-26 | 0.00 (W), 0.00 (G) | **deleterious** |
|  |  |  | R56G | 1.00 | G-58 | 0.01 | tolerated |
|  |  |  | P62S | 0.09 | S-66 | 0.00 | tolerated |
|  |  |  | **T80E** | **0.03** | V-86 | 0.00 (T), 0.00 (E) | **deleterious** |
|  |  |  | N176D | 0.14 | N181 | 0.00 | tolerated |
|  |  |  | S294G | 0.34 | S299 | 0.00 | tolerated |
|  |  |  | V300I | 0.22 | V-305 | 0.03 | tolerated |
|  |  |  | **S479R** | 0.22 | G-484 | 0.44 (S), **0.99 (R)** | **deleterious** |
|  |  |  | R920H | 0.32 | H-925 | 0.00 | tolerated |
|  | 2:128.6 | Tmem87b | S14G | 0.53 | R14 | 0.00 (S), 0.00 (G) | tolerated |
|  | 2:128.7 | Fbln7 | F10V | 0.53 | F-10 | 0.00 | tolerated |
|  |  |  | Y104H | 1 | H104 | 0.21 | tolerated |
|  |  |  | S126T | 1 | T126 | 0.07 | tolerated |
|  | 2:128.8 | Zc3h8 | V43I | 0.91 | I42 | 0.00 | tolerated |
|  | 2:128.8 | **Zc3h6** | G229A | 0.52 | G231 | 0.76 | tolerated |
|  |  |  | Y237H | 0.23 | N239 | 0.00 (Y), 0.00 (H) | tolerated |
|  |  |  | **R355S** | **0.00** | K357 | 0.15 (R), **0.97 (S)** | **deleterious** |
|  |  |  | R469H | 1.00 | H471 | 0.00 | tolerated |
|  |  |  | K482N | 1.00 | N484 | 0.00 (K), 0.00 (N) | tolerated |
|  |  |  | V556M | 0.08 | M561 | 0.00 | tolerated |
|  |  |  | R629H | 0.22 | Q634 | 0.00 (R), 0.00 (H) | tolerated |
|  |  |  | **G787R** | 0.13 | G792 | **0.93** | **deleterious** |
|  |  |  | S1002P | 1.00 | P1006 | 0.02 | tolerated |
|  |  |  | M1053T | 0.56 | S1060 | 0.00 | tolerated |
|  |  |  | I1086T | 0.46 | I1094 | 0.34 (M), 0.06 (S) | tolerated |
|  |  |  | N1094D | 0.25 | N1102 | 0.00 | tolerated |
|  |  |  | L1095M | 0.13 | V1103 | 0.00 (L), 0.01 (M) | tolerated |
|  | 2:128.9 | Polr1b | V644I | 1.00 | V644 | 0.00 | tolerated |
|  |  |  | I873V | 0.74 | I873 | 0.00 | tolerated |
|  | 2:129.0 | **Chchd5** | **R70H** | **0.03** | R70 | **1.00** | **deleterious** |
|  | 2:129.0 | Slc20a1 | E101K | 0.08 | S97 | 0.00 (E), 0.03 (K) | tolerated |
|  |  |  | S500N | 0.59 | N497 | 0.00 | tolerated |
|  |  |  | Y507C | 0.18 | Y504 | 0.00 | tolerated |
|  | 2:129.1 | **Ckap2l** | **K171R** | **0.04** | N-173 | 0.00 (K), 0.45 (R) | **deleterious** |
|  |  |  | G458V | 0.58 | ---- | ---- | tolerated |
|  |  |  | P461T | 0.72 | ---- | ---- | tolerated |
|  |  |  | V590I | 1.00 | I588 | 0.57 | tolerated |
|  | 2:130.9 | Adam33 | K85N | 1.00 | N84 | 0.79 | tolerated |
|  |  |  | T409V | 0.90 | A408 | 0.01 (T), 0.02 (V) | tolerated |
|  |  |  | R466K | 0.76 | R465 | 0.00 | tolerated |
|  | 2:130.9 | **Siglec1** | **R162G** | **0.01** | V156 | 0.19 (R), **0.85 (G)** | **deleterious** |
|  |  |  | G291R | 0.52 | Q284 | 0.00 (G), 0.00 (R) | tolerated |
|  |  |  | V292A | 0.78 | T285 | 0.00 (V), 0.00 (A) | tolerated |
|  |  |  | S507A | 1.00 | A500 | 0.12 | tolerated |
|  |  |  | N523S | 1.00 | S516 | 0.01 | tolerated |
|  |  |  | G1038S | 0.80 | G1036 | 0.08 | tolerated |
|  |  |  | T1587A | 1.00 | A1583 | 0.06 | tolerated |
|  | 2:131.0 | Hspa12b | A301S | 1.00 | S301 | 0.00 | tolerated |
|  | 2:131.1 | **Mavs** | F113V | 1.00 | L112 | 0.00 (F), 0.00 (V) | tolerated |
|  |  |  | T163A | 0.49 | T163 | 0.01 | tolerated |
|  |  |  | **A169S** | 0.30 | P170 | 0.23 (A), **0.85 (S)** | **deleterious** |
|  |  |  | S472N | 0.29 | ---- | ---- | tolerated |
|  | 2:131.4 | **Smox** | **K533N** | **0.00** | K524 | **1.00** | **deleterious** |
|  | 2:131.4 | Adra1d | A472V | 0.18 | A482 | 0.02 | tolerated |
|  | 2:139.9 | Esf1 | S90N | 0.74 | S90 | 0.00 | tolerated |
|  |  |  | K168T | 0.75 | N165 | 0.01 (K), 0.01 (T) | tolerated |
|  |  |  | E270D | 1.00 | D275 | 0.00 | tolerated |
|  | 2:140.1 | Sel1l2 | R25K | 1.00 | ---- | ---- | tolerated |
|  |  |  | L534M | 0.19 | ---- | ---- | tolerated |
|  | 2:140.2 | Macrod2 | N315S | 0.26 | ---- | ---- | tolerated |
|  |  |  | A378T | 1.00 | T335 | 0.00 | tolerated |
|  | 2:142.4 | **Kif16b** | Q759L | 0.32 | M760 | 0.00 (Q), 0.00 (L) | tolerated |
|  |  |  | F825Y | 0.76 | F825 | 0.00 | tolerated |
|  |  |  | **D929G** | **0.03** | D932 | **0.72** | **deleterious** |
|  |  |  | T937N | 1.00 | N940 | 0.01 | tolerated |
|  |  |  | A1005V | 0.19 | A1010 | 0001 | tolerated |
|  |  |  | C1019R | 0.86 | H1022 | 0.00 (C), 0.00 (R) | tolerated |
|  | 2:147.9 | Foxa2 | S272G | 0.88 | S278 | 0.00 | tolerated |
|  | 2:148.2 | **Sstr4** | **Q364R** | **0.03** | Q367 | 0.15 | **deleterious** |
|  | 2:148.3 | **Cd93** | **N264H** | **0.01** | N267 | **0.78** | **deleterious** |

Effects of each amino acid substitution in mouse proteins were predicted by SIFT (Sorting Intolerant From Tolerant) program [14]. SIFT scores show the probability that an amino acid change is damaging with a score of 0 to 1. AA substitutions with SIFT score ≤0.05 were predicted to be deleterious; substitutions with SIFT score >0.05 to be tolerated. Effects of substitutions of the residues at the equivalent position in human proteins to residues in mouse proteins were predicted by PolyPhen-2 (Polymorphism Phenotyping v2) program [15]. Where the residue in human protein differs from mouse protein, effects of substitution to both 129-type and DBA-type amino acids (shown in parentheses) were examined. PolyPhen-2 shows the probability that a mutation is damaging, ranging from 0 (benign) to 1 (damaging). ---- indicates no equivalent residue is present in human protein. Deleterious changes were shown in bold.
