# Supplementary material for: Identification of Aortic Arch-Specific Quantitative Trait Loci for Atherosclerosis by an Intercross of DBA/2J and 129S6 Apolipoprotein E-Deficient Mice
Source: PLoS One. 2015 Feb 17;10(2):e0117478. doi: 10.1371/journal.pone.0117478 (PMC4331513; doi:10.1371/journal.pone.0117478)
Supplement: S6 Table — Effects of each amino acid substitutions in mouse proteins were predicted by SIFT (Sorting Intolerant From Tolerant) program [14]. SIFT scores show the probability that an amino acid change is damaging with a score of 0 to 1. AA substitutions with SIFT score ≤0.05 were predicted to be deleterious; substitutions with SIFT score >0.05 to be tolerated. Effects of substitutions in human proteins at the equivalent residue were predicted by PolyPhen-2 (Polymorphism Phenotyping v2) program [15]. Where the residue in human protein differs from those in mice, effects of substitutions to both 129-type and DBA-type were examined. PolyPhen-2 shows the probability that a mutation is damaging, ranging from 0 (benign) to 1 (damaging). Deleterious changes were shown in bold. (DOC) [file pone.0117478.s009.doc]

**Table S6. Estimated effects of amino acid substitutions in *Aath5* candidates.**

| QTL | Chr:Mb | Gene | Substitution | SIFT | Humans | PolyPhen-2 | Effect |
| --- | --- | --- | --- | --- | --- | --- | --- |
| *Aath5* | 10:42.0 | Armc2 | A103S | 0.14 | A103 | 0.05 | tolerated |
|  | 10:43.0 | Sobp | A436P | 1.00 | P436 | 0.00 | tolerated |
|  | 10:43.5 | **Bend3** | **G435R** | 0.5 | G438 | **0.86** | **deleterious** |
|  | 10:44.0 | Aim1 | R893H | 0.40 | H913 | 0.00 | tolerated |
|  | 10:67.4 | Rtkn2 | A220T | 0.58 | A227 | 0.10 | tolerated |
|  |  |  | I331V | 0.11 | I338 | 0.00 | tolerated |
|  | 10:80.3 | **Apc2** | **R2041L** | 0.17 | R2071 | **0.99** | **deleterious** |
|  | 10:81.2 | **Zfr2** | **R624S** | 0.09 | R691 | **0.771** | **deleterious** |
|  | 10:84.8 | Btbd11 | V334A | 0.54 | A328 | 0.00 | tolerated |
|  | 10:85.5 | Syn3 | G65S | 1.00 | S-65 | 0.00 | tolerated |
|  | 10:86.3 | **Stab2** | **R151H** | **0.01** | G-143 | 0.09 (R), **1.00 (H)** | **deleterious** |
|  |  |  | K237E | 0.87 | R-229 | 0.00 (K), 0.00 (E) | tolerated |
|  |  |  | V302M | 0.06 | V294 | 0.37 | tolerated |
|  |  |  | **T382S** | 0.41 | T-374 | **0.97** | **deleterious** |
|  |  |  | I615V | 0.49 | I608 | 0.02 | tolerated |
|  |  |  | I805T | 0.55 | T798 | 0.00 | tolerated |
|  |  |  | **G864D** | **0.00** | **G 857** | **1.00** | **deleterious** |
|  |  |  | S921N | 1.00 | N913 | 0.00 | tolerated |
|  |  |  | **P1086L** | 0.08 | S1078 | 0.10 (P), **1.00 (L)** | **deleterious** |
|  |  |  | Q1208R | 1.00 | R-1200 | 0.48 | tolerated |
|  |  |  | T1311S | 0.70 | K1304 | 0.00 (T), 0.03 (S) | tolerated |
|  |  |  | **T1596M** | **0.02** | I-1581 | 0.00 (T), **0.68 (M)** | **deleterious** |
|  |  |  | S1615L | 0.37 | K-1608 | 0.00 (S), 0.16 (L) | tolerated |
|  |  |  | Q1629R | 0.75 | K1622 | 0.00 (Q), 0.00 (R) | tolerated |
|  |  |  | V1953A | 0.09 | I1945 | 0.00 (V), 0.00 (A) | tolerated |
|  | 10:87.8 | Gnptab | M206T | 1.00 | T206 | 0.00 | tolerated |
|  |  |  | W785R | 0.52 | L788 | 0.00 (Q), 0.14 (R) | tolerated |
|  |  |  | T817A | 0.76 | T825 | 0.00 | tolerated |
|  |  |  | H994D | 1.00 | D1015 | 0.11 | tolerated |
|  | 10:88.2 | Utp20 | Y1282F | 0.72 | Y1281 | 0.00 | tolerated |
|  |  |  | N1815K | 0.85 | N1814 | 0.02 | tolerated |
|  | 10:88.3 | Slc5a8 | T587N | 0.51 | T587 | 0.05 | tolerated |
|  | 10:89.0 | Slc17a8 | S581A | 0.57 | A569 | 0.00 | tolerated |
|  | 10:89.3 | Uhrf1bp1l | V346I | 1.00 | I346 | 0.00 | tolerated |
|  |  |  | I848T | 1.00 | I849 | 0.00 | tolerated |
|  |  |  | I861V | 1.00 | I862 | 0.00 | tolerated |
|  |  |  | S983A | 0.81 | V984 | 0.01 (S), 0.00 (A) | tolerated |
|  |  |  | T1017A | 0.33 | N1018 | 0.00 (T), 0.00 (A) | tolerated |
|  |  |  | N1161D | 0.40 | N1163 | 0.24 | tolerated |
|  |  |  | T1164A | 0.78 | N1166 | 0.00 (T), 0.02 (A) | tolerated |
|  |  |  | E1171D | 1.00 | D1173 | 0.00 | tolerated |
|  |  |  | T1219M | 0.13 | T1221 | 0.04 | tolerated |

Effects of each amino acid substitution in mouse proteins were predicted by SIFT (Sorting Intolerant From Tolerant) program [14]. SIFT scores show the probability that an amino acid change is damaging with a score of 0 to 1. AA substitutions with SIFT score ≤0.05 were predicted to be deleterious; substitutions with SIFT score >0.05 to be tolerated. Effects of substitutions in human proteins at the equivalent residue were predicted by PolyPhen-2 (Polymorphism Phenotyping v2) program [15]. Where the residue in human protein differs from those in mice, effects of substitutions to both 129-type and DBA-type were examined. PolyPhen-2 shows the probability that a mutation is damaging, ranging from 0 (benign) to 1 (damaging). Deleterious changes were shown in bold.
