# Supplementary material for: Identification of Aortic Arch-Specific Quantitative Trait Loci for Atherosclerosis by an Intercross of DBA/2J and 129S6 Apolipoprotein E-Deficient Mice
Source: PLoS One. 2015 Feb 17;10(2):e0117478. doi: 10.1371/journal.pone.0117478 (PMC4331513; doi:10.1371/journal.pone.0117478)
Supplement: S7 Table — Representative SNPs within and near the 123–148 Mb of Chr 2 that meet the criteria of 129 = B6 ≠ DBA and P < 1.0 × 10^5^ were selected from the eQTL data from the Hybrid Mouse Diversity Panel (HMDP) [17]. (-) or (+) in front of the distance indicates the position of the SNP is 5’ or 3’ respectively to the start of the gene. “trans” indicates the associated gene is on different chromosome. For each SNP, expression levels of the associated genes in the aorta (A) and macrophages (M) estimated by microarray analyses of the wild-type B6 and DBA strains relative to the expression of the 129 mice and the signal values of 129 expression are shown. (DOC) [file pone.0117478.s010.doc]

***Table S7. SNPs within the Aath4 interval associated with gene expressions in the aorta from Hybrid Mouse Diversity Panel.***

| Chr | SNP (Mb) | SNP name | Distance (Mb) | Gene symbol | Chr | Gene start (Mb) | P | DBA/129 (A) | B6/129 (A) | Level (A) | DBA/129 (M) | B6/129 (M) | Level (M) |
| --- | --- | --- | --- | --- | --- | --- | --- | --- | --- | --- | --- | --- | --- |
| 2 | 125.2 | rs29767552 | +0.42 | Secisbp2l | 2 | 125.6 | 5.37E-07 | 0.9 | 0.9 | 699 | 1.0 | 1.1 | 584 |
| 2 | 125.3 | rs27421939 | trans | Kcne4 | 1 | 78.8 | 9.27E-06 | 2.1 | 1.5 | 172 | 1.3 | 0.6 | 18 |
| 2 | 125.5 | rs27435685 | trans | Mtor | 4 | 147.8 | 4.69E-06 | 1.0 | 1.2 | 850 | 1.1 | 1.0 | 615 |
| 2 | 125.5 | rs27435685 | trans | Cd24a | 10 | 43.3 | 3.26E-06 | **0.7^a^** | 0.7 | 101 | 0.7 | 0.5 | 190 |
| 2 | 125.6 | rs3725315 | +1.19 | Sppl2a | 2 | 126.8 | 6.42E-12 | 1.2 | 1.1 | 736 | 1.6 | 0.9 | 1475 |
| 2 | 125.8 | rs27449091 | -0.19 | Dtwd1 | 2 | 126.0 | 9.24E-30 | 1.1 | 0.9 | 158 | 1.1 | 0.9 | 170 |
| 2 | 126.7 | rs29561237 | +0.35 | Ciao1 | 2 | 127.1 | 3.53E-10 | 0.9 | 1.0 | 661 | 1.4 | 0.9 | 556 |
| 2 | 126.9 | rs27466128 | +1.46 | Eid1 | 2 | 125.5 | 1.12E-15 | **1.8^a^** | 1.3 | 125 | 1.4 | 1.2 | 59 |
| 2 | 127.6 | rs33372274 | +0.01 | Nphp1 | 2 | 127.6 | 6.07E-11 | **1.3^a^** | 1.0 | 147 | 1.3 | 1.0 | 88 |
| 2 | 128.0 | rs27431979 | +0.05 | Bcl2l11 | 2 | 128.0 | 2.16E-10 | 0.7 | 0.8 | 62 | 0.8 | 2.0 | 262 |
| 2 | 129.0 | rs13469412 | +0.00 | Chchd5 | 2 | 129.0 | 1.95E-10 | 1.0 | 0.9 | 192 | 1.2 | 1.0 | 323 |
| 2 | 131.2 | rs29582786 | trans | Rabl3 | 16 | 37.5 | 9.85E-06 | 1.2 | 1.0 | 178 | 1.8 | 0.9 | 403 |
| 2 | 131.4 | rs27259068 | -0.34 | Spef1 | 2 | 131.0 | 8.16E-19 | **0.5^a^** | 1.3 | 175 | 0.7 | 1.2 | 87 |
| 2 | 140.7 | rs33556324 | trans | Nub1 | 5 | 24.2 | 1.74E-07 | 0.8 | 1.0 | 22 | 1.1 | 1.6 | 763 |
| 2 | 140.7 | rs33109441 | +0.75 | Ndufaf5 | 2 | 140.0 | 5.29E-10 | 1.3 | 0.9 | 53 | 0.9 | 0.7 | 248 |
| 2 | 148.1 | rs27265041 | trans | Tle1 | 4 | 71.9 | 9.31E-07 | 1.0 | 1.0 | 7 | 1.0 | 1.2 | 46 |
| 2 | 148.1 | rs27265041 | trans | Hoxb4 | 11 | 96.2 | 6.12E-06 | 1.1 | 1.0 | 69 | 0.7 | 1.0 | 88 |
| 2 | 148.2 | rs27297236 | -0.19 | Mapre1 | 2 | 153.6 | 6.04E-14 | 0.9 | 0.9 | 679 | 1.3 | 1.0 | 691 |

Representative SNPs within and near the 123–148 Mb of Chr 2 that meet the criteria of 129 = B6 ≠ DBA and *P* < 1.00E-05 were selected from the eQTL data from the Hybrid Mouse Diversity Panel (HMDP) [17]. (-) or (+) in front of the distance indicates the position of the SNP is 5’ or 3’ respectively to the start of the gene. “trans” indicates the associated gene is on a different chromosome. For each SNP, expression levels of the associated genes in the aorta (A) and macrophages (M) estimated by microarray analyses of the wild-type B6 and DBA strains relative to the expression of the 129 mice and the signal values of 129 expression are shown. ^a^*P* < 0.05.
