# Supplementary material for: Identification of Aortic Arch-Specific Quantitative Trait Loci for Atherosclerosis by an Intercross of DBA/2J and 129S6 Apolipoprotein E-Deficient Mice
Source: PLoS One. 2015 Feb 17;10(2):e0117478. doi: 10.1371/journal.pone.0117478 (PMC4331513; doi:10.1371/journal.pone.0117478)
Supplement: S8 Table — Representative SNPs within and near the 30–101 Mb intervals of Chr 10 and associated genes that meet the criteria of 129 = B6 ≠ DBA and P < 1.0 × 10^5^ were selected from the eQTL data from the Hybrid Mouse Diversity Panel (HMDP) [17]. For each SNP, expression levels of associated genes in the aorta (A) and macrophages (M) estimated by microarray analyses of the wild-type 129, B6 and DBA strains were indicated. (DOC) [file pone.0117478.s011.doc]

***Table S8. SNPs within the Aath5 interval associated with gene expressions in the aorta from Hybrid Mouse Diversity Panel.***

| Chr | SNP  (Mb) | SNP name | Distance  (Mb) | Gene symbol | Chr | Start  (Mb) | P | DBA/129  (A) | B6/129  (A) | Level  (A) | DBA/129  (M) | B6/129  (M) | Level  (M) |
| --- | --- | --- | --- | --- | --- | --- | --- | --- | --- | --- | --- | --- | --- |
| 10 | 68.6 | rs29334455 | +17.8 | Stab2 | 10 | 86.5 | 2.65E-07 | **18.0^c^** | 1.4 | 16 | 11.0 | 1.4 | 18 |
| 10 | 69.0 | rs29360408 | trans | Serpinb1a | 13 | 32.9 | 4.51E-06 | 0.8 | 0.6 | 54 | 0.5 | 0.5 | 177 |
| 10 | 69.0 | rs29351715 | trans | Socs3 | 11 | 117.8 | 3.97E-06 | 1.0 | 1.0 | 10 | 1.3 | 0.7 | 20 |
| 10 | 85.0 | rs29327398 | Trans | Mst1r | 9 | 107.8 | 4.55E-07 | 1.4 | 1.2 | 60 | 0.9 | 0.8 | 110 |
| 10 | 85.0 | rs29327412 | +4.23 | Actr6 | 10 | 89.2 | 1.02E-06 | **0.7^a^** | 1.1 | 211 | 0.9 | 1.0 | 154 |
| 10 | 85.0 | rs29364260 | trans | Tbc1d8 | 1 | 39.5 | 2.36E-06 | 1.1 | 0.9 | 144 | 1.4 | 0.8 | 128 |
| 10 | 85.7 | rs29319047 | +0.79 | Stab2 | 10 | 86.5 | 5.48E-17 | **18.0^c^** | 1.4 | 16 | 11.0 | 1.4 | 18 |
| 10 | 87.5 | rs29384472 | +4.69 | Nedd1 | 10 | 92.2 | 4.47E-07 | 0.9 | 1.1 | 64 | 1.0 | 1.0 | 89 |
| 10 | 87.6 | rs29320710 | -5.36 | Hal | 10 | 93.0 | 2.41E-07 | **0.2^c^** | **0.1^c^** | 157 | 0.3 | 0.05 | 3238 |
| 10 | 87.9 | rs29339575 | +0.04 | Chpt1 | 10 | 88.0 | 9.60E-05 | 0.7 | 1.2 | 929 | 2.1 | 1.1 | 124 |
| 10 | 87.9 | rs29339575 | +0.09 | Gnptab | 10 | 87.8 | 1.78E-10 | **0.8^a^** | 1.0 | 481 | 1.0 | 1.1 | 1082 |
| 10 | 88.0 | rs29327022 | trans | 9530068E07Rik | 11 | 52.2 | 2.40E-09 | 0.9 | 1.0 | 621 | 1.0 | 0.8 | 656 |
| 10 | 88.0 | rs29330703 | trans | D19Bwg1357e | 19 | 27.5 | 7.37E-07 | 1.0 | 0.9 | 205 | 1.3 | 0.8 | 393 |
| 10 | 88.1 | rs29378667 | trans | Med8 | 4 | 118.1 | 2.57E-06 | 1.2 | **1.3^a^** | 67 | 0.9 | 0.8 | 189 |
| 10 | 88.1 | rs4228381 | +6.65 | Cradd | 10 | 94.8 | 2.91E-06 | 1.0 | 1.0 | 97 | 1.3 | 1.1 | 142 |
| 10 | 88.2 | rs29335856 | trans | Pxmp2 | 5 | 110.7 | 4.29E-06 | 1.4 | 1.5 | 54 | 1.0 | 1.0 | 57 |
| 10 | 88.6 | rs29337956 | -1.93 | Ikbip | 10 | 90.5 | 2.17E-06 | 1.0 | 0.9 | 753 | 1.3 | 0.8 | 186 |

Representative SNPs within and near the 30-101Mb intervals of Chr 10 and associated genes that meet the criteria of 129 = B6 ≠ DBA and *P* < 1.00E-05 were selected from the eQTL data from the Hybrid Mouse Diversity Panel (HMDP) [17]. For each SNP, expression levels of associated genes in the aorta (A) and macrophages (M) estimated by microarray analyses of the wild-type 129, B6 and DBA strains were indicated. ^a^*P* < 0.05, ^c^*P* < 0.001.
