# Supplementary material for: Identification of Aortic Arch-Specific Quantitative Trait Loci for Atherosclerosis by an Intercross of DBA/2J and 129S6 Apolipoprotein E-Deficient Mice
Source: PLoS One. 2015 Feb 17;10(2):e0117478. doi: 10.1371/journal.pone.0117478 (PMC4331513; doi:10.1371/journal.pone.0117478)
Supplement: S9 Table — Genes with DBA-unique sequences (DBA ≠ B6, 129) within and near the interval of 123–148 Mb are shown. For each gene, expression ratios in the aorta and macrophages, expression levels in 129, amino acid (AA) differences (B6, 129-position-DBA), associations with atherosclerosis indicated by GWAS data, and phenotypes of knockout or mutant mice are shown. Ratios between two strains that show significant difference in the expression are bolded. AA substitutions that are predicted to be deleterious by SIFT and/or Polyphen2 programs are bolded (see S5 Table). a P < 0.05. (DOC) [file pone.0117478.s012.doc]

**Table S9. Haplotype analysis of *Aath4* on Chr 2.**

| Gene | Mb | Haplotype | Aortic expression | | | Macrophage expression | | | AA substitution | Human mutation, GWAS (-log_10_P) | KO / mutant mouse phenotype |
| --- | --- | --- | --- | --- | --- | --- | --- | --- | --- | --- | --- |
|  |  |  | DBA/129 | B6/129 | Level | DBA/129 | B6/129 | Level |  |  |  |
| Fbn1 | 125.1 | 129, B6 ≠ DBA, C3H | 1.1 | 1.5 | 1684 | 0.9 | 0.2 | 127 | A427V, **D492N** | Cholesterol (10.0), Aortic aneurysm (12.2) | Right ventricle dilation |
| Cep152 | 125.4 | 129, B6 ≠ DBA, C3H | 1.0 | 0.9 | 34 | 1.0 | 1.1 | 102 | **K979T, R1307G** and 16 others |  |  |
| Eid1 | 125.5 | 129, B6 ≠ DBA, C3H | **1.8^a^** | 1.3 | 125 | 1.4 | 1.2 | 59 |  | apoE level (8.4) |  |
| Shc4 | 125.5 | 129, B6 ≠ DBA, C3H | 1.2 | 1.3 | 29 | 1.1 | 0.9 | 22 | A364T | Heart failure (3.0), ApoE level (8.4) |  |
| Secisbp2l | 125.7 | 129, B6 ≠ DBA, C3H | 0.9 | 0.9 | 699 | 1.0 | 1.1 | 584 | **D206Y**, A601T |  |  |
| Galk2 | 125.7 | 129, B6 ≠ DBA, C3H | 0.9 | 1.0 | 140 | 0.9 | 0.8 | 10 | P117R, M219V, **R299K** |  |  |
| Dtwd1 | 126.0 | 129, B6 ≠ DBA, C3H | 1.1 | 0.9 | 158 | 1.1 | 0.9 | 170 | S25P, T28A, **E33G**, D117N, E184K | Antidepressant effect (6.3) |  |
| Atp8b4 | 126.1 | 129, B6 ≠ DBA, C3H | 1.0 | 1.0 | 12 | 1.2 | 1.2 | 367 | I424S, N540S, S726N, D737E, A746S | HDL (5.2), CAD (4.3), Stroke (3.3) |  |
| Slc27a2 | 126.4 | 129, B6 ≠ DBA, C3H | **0.2^a^** | 1.7 | 412 | 1.0 | 1.1 | 16 | L529I, A547S |  | Reduced very long fatty acid degradation |
| Hdc | 126.4 | 129, B6 ≠ DBA, C3H | 0.9 | 0.9 | 24 | 0.6 | 0.3 | 53 | **K177T** | Stroke (3.9) | Abnormal mast cell morphology, Decreased plaques in *Hdc^-/-^Apoe^-/-^* mice |
| Gabpb1 | 126.5 | 129, B6 ≠ DBA, C3H | 1.0 | 1.0 | 84 | 1.2 | 0.9 | 118 |  | Body weight (4.8) | Embryonic lethal |
| Ap4e1 | 126.8 | 129, B6 ≠ DBA, C3H | 0.9 | 0.9 | 178 | 1.1 | 1.0 | 245 | **Q448H,** F847L  T1051A | Cerebral palsy syndrome | Anemia, Iron deficiency |
| Dusp2 | 127.2 | 129, B6 ≠ DBA, C3H | 0.9 | 1.0 | 46 | 1.0 | 1.6 | 66 | **A103T** |  | Abnormal mast cell physiology |
| Adra2b | 127.2 | 129, B6 ≠ DBA, C3H | 1.0 | 1.1 | 27 | 1.0 | 1.1 | 28 |  |  | Abnormal vasoconstriction, lung development |
| Gpat2 | 127.3 | 129, B6 ≠ DBA, C3H | 1.2 | 0.8 | 12 | 1.0 | 1.2 | 13 | **A238V** |  | Lipodystrophy |
| Prom2 | 127.4 | 129, B6 ≠ DBA, C3H | 0.9 | 0.8 | 26 | 1.0 | 1.3 | 24 | **R411C, V495A** |  |  |
| Zfp661 | 127.4 | 129, B6 ≠ DBA, C3H | 1.0 | 1.0 | 38 | 0.8 | 1.3 | 61 | **A2S,** I74M, G368S |  |  |
| Mall | 127.5 | 129, B6 ≠ DBA, C3H | **0.5^a^** | 0.9 | 350 | 0.9 | 1.6 | 25 |  |  |  |
| Nphp1 | 127.5 | 129, B6 ≠ DBA, C3H | **1.3^a^** | 1.0 | 147 | 1.3 | 1.0 | 88 | D217E, **L540M,** S590A, A662T | Juvenile nephronophthisis, Cystic kidney | Retinal degeneration |
| Bcl2l11 | 128.0 | 129, B6 ≠ DBA, C3H | 0.7 | 0.8 | 62 | 0.8 | 2.0 | 262 |  | CAD (4.2), Monocytes(6.4) | Vasculitis, Increased lymphocytes |
| Mertk | 128.5 | 129, B6 ≠ DBA, C3H | 1.0 | **1.4^a^** | 784 | 0.8 | 1.7 | 782 | **W25G**, **T80E**, **S479R**, and 6 others | Retinitis pigmentosa.  Stroke (3.3) | Abnormal retinal vascular morphology, Impaired platelet aggregation, Increased necrosis in *Mertk^KD^Apoe^-/-^* plaques |
| Fbln7 | 128.7 | 129, B6 ≠ DBA, C3H | 0.9 | 0.6 | 358 | 0.8 | 1.1 | 40 | F10V, Y104H, S126T | Heart rate (4.3) |  |
| Zc3h6 | 128.8 | 129, B6, C3H ≠ DBA | 0.9 | 0.7 | 88 | 0.9 | 1.0 | 1 49 | **R355S, G787R**, and 7 others | Spinal bifida |  |
| Chchd5 | 129.0 | 129, B6, C3H ≠ DBA | 1.0 | 0.9 | 192 | 1.2 | 1.0 | 323 | **R70H** | Body fat distribution (4.0) |  |
| Ckap2l | 129.1 | 129, B6, C3H ≠ DBA | 0.9 | 1.2 | 21 | 0.9 | 0.9 | 38 | **K171R,** G458V, P461T, V590I | Endometriosis (5.5) |  |
| Adam33 | 130.9 | 129, B6, C3H ≠ DBA | 0.8 | 1.1 | 176 | 0.7 | 2.7 | 47 | K85N, T409V, R466K | BP (4.1) | Normal phenotype |
| Siglec1 | 130.9 | 129, B6, C3H ≠ DBA | 1.5 | 1.4 | 47 | 0.6 | 2.2 | 787 | **R162G**, and 6 others |  | Abnormal B cell number |
| Hspa12b | 131.0 | 129, B6, C3H ≠ DBA | 1.0 | 1.0 | 238 | 1.0 | 1.1 | 20 | A301S | CVD (5.3) |  |
| Spef1 | 131.0 | 129, B6, C3H ≠ DBA | **0.5^a^** | 1.3 | 175 | 0.7 | 1.2 | 87 |  | CAD (4.2) |  |
| Rnf24 | 131.1 | 129, B6, C3H ≠ DBA | 1.2 | 1.2 | 276 | 1.1 | 0.7 | 102 |  | BP (5.4) |  |
| Smox | 131.3 | 129, B6, C3H ≠ DBA | 1.2 | 1.2 | 232 | 1.0 | 1.0 | 261 | **K533N** | Hematocrit (5.0) |  |
| Adra1d | 131.4 | 129, B6, C3H ≠ DBA | 1.3 | 1.1 | 755 | 1.0 | 1.4 | 16 | A472V | Uric acid (7.1), Respiratory function (6.5) | Hypotension |
| Plcb1 | 134.6 | 129, B6, C3H ≠ DBA | 0.9 | 0.8 | 449 | 0.8 | 0.7 | 180 |  | ApoB (5.2), Stroke (3.0) | Hyperactive |
| Plcb4 | 135.5 | 129, B6, C3H ≠ DBA | 1.0 | 0.9 | 1671 | 0.8 | 0.3 | 101 |  | BW (5.7), Medial thickness (4.2), Leukocyte counts (9.5), HDL (4.2) | Hypoactive, Impaired coordination |
| Tasp1 | 140.0 | 129, B6, C3H ≠ DBA | 0.9 | 0.9 | 91 | 0.9 | 1.0 | 77 |  | MI (3.8) | Abnormal vertebrae morphology |
| Macrod2 | 140.2 | 129, B6, C3H ≠ DBA | 1.2 | 1.2 | 20 | 1.1 | 0.8 | 18 | Q55R, L63F, V66F, K76R, R77Q, N315S, A378T | Coronary artery calcification (4.4), BMI (6.8), platelet count (6.4) |  |
| Kif16b | 142.4 | 129, B6, C3H ≠ DBA | 0.8 | 1.0 | 351 | 1.0 | 1.2 | 436 | Q759L, F825Y, **D929G**, T937N, A1005V, C1019R | Glucose(6.0) | Embryonic lethality, Absence of epiblast |
| Pcsk2 | 143.4 | 129, B6, C3H ≠ DBA | 1.3 | 1.1 | 13 | 1.0 | 1.0 | 12 |  | Carotid intimal medial thickness (4.5), Menarche (7.5) | Impaired processing of neuroendocrine hormones, Hypertension |
| Foxa2 | 147.9 | 129, B6, C3H ≠ DBA | 1.0 | 0.9 | 32 | 1.0 | 1.3 | 27 | S272G |  | Embryonic lethal in homozygotes, Abnormal cardiovascular development in heterozygotes |
| Sstr4 | 148.2 | 129, B6, C3H ≠ DBA | 1.0 | 1.1 | 28 | 1.0 | 1.3 | 25 | **Q346R** |  | Pulmonary edema |
| Thbd | 148.2 | 129, B6, C3H ≠ DBA | 0.8 | 1.1 | 1110 | 1.0 | 1.0 | 257 |  | Osteoporosis (12.5) | Abnormal leukocyte adhesion, Increased MI size |
| Cd93 | 148.3 | 129, B6, C3H ≠ DBA | 1.1 | 1.2 | 489 | 0.9 | 1.3 | 1667 | **N264H** |  | Impaired macrophage phagocytosis |
| Cst3 | 148.7 | 129, B6 ≠ DBA, C3H | **1.2^a^** | 1.0 | 3987 | 1.2 | 1.0 | 3759 |  | Kidney disease (137.7) | Thinning of tunica media and increased SM cell in atherosclerotic plaques in *Cst3^-/-^;Apoe^-/-^* mice |

Genes with DBA-unique sequences (DBA ≠ B6, 129) within and near the interval of 123–148 Mb are shown. For each gene, expression ratios in the aorta and macrophages, expression levels in 129, amino acid (AA) differences (B6, 129-position-DBA), associations with atherosclerosis indicated by GWAS data, and phenotypes of knockout or mutant mice are shown. Ratios between two strains that show significant difference in the expression are bolded. AA substitutions that are predicted to be deleterious by SIFT and/or Polyphen2 programs are bolded (see table S5). ^a^*P* < 0.05.
