# Supplementary material for: Identification of Aortic Arch-Specific Quantitative Trait Loci for Atherosclerosis by an Intercross of DBA/2J and 129S6 Apolipoprotein E-Deficient Mice
Source: PLoS One. 2015 Feb 17;10(2):e0117478. doi: 10.1371/journal.pone.0117478 (PMC4331513; doi:10.1371/journal.pone.0117478)
Supplement: S10 Table — Genes with DBA-unique sequences (DBA ≠ B6, 129) within and near the interval of 30–101 Mb are shown. For each gene, expression ratios in the aorta and macrophages, expression levels in 129, amino acid (AA) differences (B6, 129-position-DBA), associations with atherosclerosis indicated by GWAS data, and phenotypes of knockout or mutant mice are shown. Ratios between two strains that show significant difference in the expression are bolded. AA substitutions that are predicted to be deleterious by SIFT and/or Polyphen2 programs are bolded (see S6 Table). a P < 0.05, b P < 0.01, c P < 0.001. (DOC) [file pone.0117478.s013.doc]

**Table S10. Haplotype analysis of *Aath5* on Chr 10.**

| Gene | Mb | Haplotype | Aortic expression | | | Macrophage expression | | | AA substitution | Human mutation,  GWAS (-log_10_P) | KO / mutant mice phenotype |
| --- | --- | --- | --- | --- | --- | --- | --- | --- | --- | --- | --- |
|  |  |  | DBA/129 | B6/129 | Level | DBA/129 | B6/129 | Level |  |  |  |
| Foxo3 | 42.2 | 129 ≠ B6, DBA | 1.0 | 1.3 | 187 | 1.3 | 1.9 | 174 |  | IGF1 (6.3), HDL (6.8) | Premature death, abnormal blood vessels |
| Bend3 | 43.5 | 129 ≠ B6, DBA | 1.3 | 1.2 | 103 | 1.1 | 0.8 | 136 | **G435R** |  |  |
| Aim1 | 44.0 | 129 ≠ B6, DBA | 1.0 | 1.0 | 18 | 1.5 | 1.6 | 266 | R893H | Stroke (5.0) |  |
| Mcm9 | 53.6 | 129 ≠ B6, DBA | 1.0 | 1.0 | 55 | 1.1 | 1.0 | 136 | S93A |  | Spontaneous chromosome breakage |
| Rtkn2 | 67.4 | 129, B6 ≠ DBA | 0.9 | 0.7 | 7 | 1.1 | 1.2 | 19 | A220T, I331V | HDL (9.9) |  |
| Arid5b | 67.6 | 129, DBA ≠ B6 | 0.9 | 1.2 | 1282 | 1.3 | 1.0 | 623 |  | Leukemia (18.2), Rheumatoid arthritis (17.2) | Postnatal death, Abnormal SM morphology |
| C10orf107 | 67.9 | 129, B6 ≠ DBA | 1.0 | 1.2 | 85 | 1.0 | 0.7 | 24 |  | Diastolic BP (17.7), Pulse(10.0) |  |
| Tmem26 | 68.2 | 129, B6 ≠ DBA | 0.8 | 1.5 | 167 | 1.4 | 2.5 | 105 |  | Insulin (4.6) |  |
| Cdk1 | 68.8 | 129, B6 ≠ DBA | 0.8 | 1.0 | 75 | 1.2 | 0.7 | 136 |  | MCP1 level (6.5) | Embryonic lethal |
| Tcp11l2 | 84.0 | 129, B6 ≠ DBA | 1.0 | **1.2^a^** | 231 | 1.2 | 1.9 | 115 |  |  |  |
| Rfx4 | 84.2 | 129, B6 ≠ DBA | 1.1 | 1.1 | 16 | 0.9 | 0.8 | 15 |  |  | Heterozygotes die prematurely Altered response to MI |
| Btbd11 | 84.8 | 129, B6 ≠ DBA | 1.1 | **1.2^b^** | 36 | 1.0 | 0.8 | 20 | V334A |  |  |
| Nt5dc3 | 86.2 | 129, B6 ≠ DBA | **1.4^a^** | 1.0 | 270 | 1.2 | 1.0 | 275 |  | Acute myeloid leukemia |  |
| Stab2 | 86.3 | 129, B6 ≠ DBA | **18.0^c^** | 1.4 | 16 | **11.0** | 1.4 | 18 | **R151H**, **G864D**, **T1596M**, and 12 others | Coronary restenosis (7.0), CAD (5.0), Stroke (3.3) | Increased plasma hyaluronan |
| Ascl1 | 87.0 | 129, B6 ≠ DBA | 0.9 | 0.9 | 19 | 0.9 | 0.9 | 17 |  | C-reactive protein (10.7) | Neonatal death, Abnormal neuron apoptosis |
| Igf1 | 87.3 | 129, B6 ≠ DBA | 0.7 | 1.6 | 297 | 0.8 | 0.8 | 2177 |  | Insulin resistance (8.7) | Abnormal glucose metabolism, Premature death, |
| Pmch | 87.5 | 129, B6 ≠ DBA | 1.1 | 0.9 | 11 | 1.0 | 1.1 | 11 |  |  | Regulation of heart rate, BP |
| Ccdc53 | 87.7 | 129, B6 ≠ DBA | 1.0 | 1.0 | 819 | 1.0 | 1.1 | 1223 |  |  | Female infertility |
| Gnptab | 87.8 | 129, B6 ≠ DBA | **0.8^b^** | 1.0 | 485 | 0.9 | 1.0 | 1038 | M206T, W785R, T817A, H994D | Mucolipidosis | Abnormal peptide metabolism, Growth retardation |
| Chpt1 | 87.9 | 129, B6 ≠ DBA | 0.7 | 0.9 | 919 | 1.8 | 1.2 | 115 |  | VLDL (4.3) |  |
| Arl1 | 88.2 | 129, B6 ≠ DBA | 0.9 | 1.0 | 1285 | 1.1 | 0.8 | 1125 |  |  | Postnatal death, Respiratory distress |
| Utp20 | 88.2 | 129, B6 ≠ DBA | 1.0 | 0.8 | 192 | 1.1 | 0.9 | 325 | Y1281F, N1815K | Atherosclerosis (5.2) |  |
| Slc5a8 | 88.3 | 129, B6 ≠ DBA | 1.6 | 1.0 | 9 | 0.9 | 1.3 | 8 | T587N | HDL (9.8) | Abnormal urine homeostasis |
| Actr6 | 89.2 | 129, B6 ≠ DBA | **0.7^a^** | 1.1 | 211 | 0.9 | 1.0 | 154 | F263S |  |  |
| Hal | 93.5 | 129, DBA ≠ B6 | **0.2^c^** | **0.1^c^** | 157 | 0.3 | 0.1 | 3238 |  | Histidinemia | Increased urinary histidine |

Genes with DBA-unique sequences (DBA ≠ B6, 129) within and near the interval of 30-101 Mb are shown. For each gene, expression ratios in the aorta and macrophages, expression levels in 129, amino acid (AA) differences (B6, 129-position-DBA), associations with atherosclerosis indicated by GWAS data, and phenotypes of knockout or mutant mice are shown. Ratios between two strains that show significant difference in the expression are bolded. AA substitutions that are predicted to be deleterious by SIFT and/or Polyphen2 programs are bolded (see table S6). ^a^*P* < 0.05, ^b^*P* < 0.01, ^c^*P* < 0.001.
